# TPAAS: Trustworthy privacy-preserving anonymous authentication scheme for online trading environment

**DOI:** 10.1371/journal.pone.0307738

**Published:** 2024-11-18

**Authors:** Arun Sekar Rajasekaran, Azees Maria, Jaime Lloret, Suresh Dannana

**Affiliations:** 1 Department of ECE, SR University, Warangal, Telangana, India; 2 School of Computer Science and Engineering, VIT-AP University, Amaravathi, Andhra Pradesh, India; 3 Instituto de Investigación para la Gestión Integrada de Zonas Costeras, Universitat Politècnica de València, Valencia, Spain; 4 Department of ECE, GMR Institute of Technology, Rajam, Andhra Pradesh, India; Jinan University, CHINA

## Abstract

In recent years with the improvement of information communication technology (ICT) and wireless communication, Online Trading Environment (*OTE*) has become a popular E-commerce platform to connect sellers and buyers in an efficient way. As, *OTE*’s are increasing in a wider range, the authentication and verification of entities in *OTE* network becomes a challenging task. Although, some authentication schemes exist in *OTE*’s, they have flaws such as account creation delays, authentication delays, communication cost and user privacy. In this work, a trustworthy and secure anonymous authentication scheme is proposed to prevent malicious users to enter into the *OTE* network. In addition, our proposed scheme provides conditional privacy to users until they maintain a genuine relationship with other entities without compromising. If any dispute occurs, then the system will revoke the access of that particular entity. Moreover, the security and performance analysis in this work concludes that our scheme ensures a secure interface to provide sustainable trading experience to users by taking less computation cost and communication delay when compared to other existing authentication schemes.

## 1. Introduction

With the advancement of the internet, many activities are now carried out via online. Moreover, trading of goods and services are performed through online in a single click. The Online Trading Environment (*OTE*)’s are growing at a faster rate and analytics estimated that these *OTE*’s provides 40% of the global online retail market during 2020. *OTE* allows companies to internationalize its business and it also allows the consumers to easily find their necessary products by making deals with sellers through online after verifying their identities at respective E-commerce platform [1]. Progressively buyers (*B*_*i*_) are given with more preference to make purchase using *OTE* by skipping the excursion to the store. By using *OTE*, buyer is no longer confined to buy the products which are available in one country or in one town or in one store, but he/she can access the *OTE* system from anywhere at any time [2]. As internet has no boundaries, any user can access goods and services from any part of the globe.

*OTE* has emerged as a convincing and accepted business standard by using which sellers and buyers can communicate each other to participate in the buying and selling process. Today most of the companies are incorporating the E-commerce concept at some level of their businesses [3]. In addition, there are some retail stores who expand their market through *OTE* having both virtual store and a physical store. So, these *OTE*’s become a convenient platform for sellers to increase their profits and it became a popular way of purchasing goods for the buyers. The only requirement for buyer is PC with internet access.

Although, *OTE* is offering lot of ease and convenience of shopping, there are some disadvantages like lack of anonymity and trust among users [4]. In the exiting *OTE*, a seller can create an account by giving his fake credentials [5]. Moreover, there is no physical interaction between sellers and buyers, so buyers cannot see the product physically before purchasing it. Therefore, it may cause consumers to obtain faulty goods [6, 7]. In addition, some malicious users can enter the network using authorized user details to commit fraud (impersonation attack).

Hence, the essential security requirement of *OTE* is data integrity. In *OTE*, the online trading platform (*OTP*) which is a trusted component / entity in the *OTE* network which preserves the data securely [8] and monitor the behavior of mediators (*M*_*i*_) and users (*U*_*i*_) in a proper way.

In *OTE*, entities communicate through the wireless medium [9]. Therefore, it is essential to ensure fundamental security requirements, such as availability, integrity, confidentiality, user authentication, and privacy in e-commerce [10, 11]. Hence, to ensure a trustworthy anonymous authentication with minimum complexity and simple privacy revoking process, a secure, efficient and trustworthy anonymous authentication with conditional privacy preserving scheme for *OTE* is proposed to contribute sustainable trading [12]. The *OTE* is developed to address the following essential security requirements: 1) The *OTP* offers conditional privacy to the mediators and users [13]. 2) Mediators and users can generate anonymous certificates individually without storing them in the database to preserve their privacy [14]. 3) If any misbehavior of any entity occurs, the *OTP* can track the identity and revoke the access of malicious user by revealing its true identity [15].

The main contribution of our proposed system to deal with the above-mentioned challenges in *OTE* are,

- To develop an anonymous authentication scheme for both *U*_*i*_ and *M*_*i*_ which has less computational complexity to minimize the computational delay.
- To provide data integrity at the minimum certificate and signature verification cost.
- To minimize the communication cost between the user and mediator.
- To provide conditional user privacy to revoke the access of any misbehaving *U*_*i*_ or *M*_*i*_ in *OTE* system.

The rest of this article is summarized as follows. Related works are discussed in section 2. In section 3, system overview is demonstrated. The proposed *OTE* system is explained in section 4. In section 5 and 6, the security and performance analysis of the proposed *OTE* are described. Finally, section 7 concludes this work.

## 2. Related work

The majority of research in the field of online security and privacy has focused on authentication to ensure security. Although, there exist some authentication techniques which prevents malicious users to enter into network, they consume a lot of computation delay and in these techniques, there is possibility of user’s true identity can be disclosed. Azad, M. A. *et al*. [16] proposed a decentralized reputation system to provide the privacy for the consumer’s feedback values. These feedback values are given by consumer about a particular retailer whom he met on the online market place for the purpose of buying a product. Here, the authors used decentralized zero-knowledge proof primitives and homographic cryptographic methods while creating the privacy preserving system known as “PrivBox”. Moreover, there will be no trusted third party to provide the privacy for the consumers and to enable retailers and consumers to authenticate each other. In addition, the authors used encrypted exchange of feedback values and zero-knowledge proof of knowledge to provide well- formedness of encrypted values. Though this system provides privacy, it is not open distributed and not possible to verify the correctness of the feedback values in future. While giving feedback a malicious or compromised consumer may give false feedback about a retailer and which leads to decrement in the score of that particular retailer. Moreover, there is no particular software tool which allows retailers to see their scores frequently.

Hampiholi *et al*. [17] proposed a cryptographic webshopping scheme to provide privacy for the sensitive information of both buyers and sellers, where this scheme is based on attribute credentials where attributes represent pieces of data. Here, the purchasers reveal only the required information at each stage of shopping so that the bank that processes the payment don’t know the information of product, buyer’s shipping address and account details. The work suffers from unlinkability, since an adversary may inject false information at the stage of transaction. For instance, an adversary may modify the shipping address after the payment has been done by the actual buyer. Moreover, this system needs different layers of interfaces while doing transactions and computation cost also increases.

Niu, C. *et al*. [18] proposed an efficient, privacy-preserving and verifiable online auction scheme where they used novel protocol and Paillier homomorphic encryption for providing privacy and for verifiability of transactions. Though it provides low computation and communication costs with less storage overheads, it doesn’t perform any authentication process so that malicious user also can enter into network and can damage the auction procedure. There is no dynamic pricing scheme, so advertisers who lost in auction cannot win in next rounds thereby decreasing their probability to win in auction.

Helsloot *et al*. [19] proposed an online behavioral advertising scheme which provides privacy for the user’s data. Moreover, the authors used homographic encryption with a machine learning method to process user’s data in encrypted form thereby providing privacy. Though it provides privacy preserving online behavioral advertising protocol, there is less safety to user’s data because it may go into the hands of malicious user. As there are no authentication procedures, an intruder can enter into the network and give advertisements which disturbs the user.

Ranganthan *et al*. [20] designed a decentralised marketplace application using truffle development framework to achieve privacy for user’s data, to decrease sudden blocking of user’s account in online market places and to allow a particular user to both sell and buy the products. Here, the application will function in ethereum network, where the user’s data are taken by the interface and given to the ethereum network. Though, it has less transaction runtime, there is no mediators in the network and the seller/buyer can’t find their required product and the user’s real identity may be revealed to adversaries because of lack of anonymity.

Jiang *et al*. [21] proposed a privacy preserving business protocol using private smart contract in the negotiation phase. This system allows sellers and buyers to make deals without revealing their real credentials like name, address and phone number. Moreover, the authors used a zero-knowledge proof to guarantee the legitimacy of the user. Though this system achieves both proof of ownership and privacy protection, it takes more time and computation cost as the transactions are stored in blocks. Moreover, there is no authentication procedures and malicious users can enter into the network.

Scaria *et al*. [22] proposed an E-commerce application to perform transactions where they provide more security for user’s account by using three factor authentication i.e., the authors performed three types of authentications to login into user’s account. They are combination of using one time password, using noisy password and performing biometric or palm-vein scan. Though, this approach provides a lot of security to user’s account and it is not possible for an attacker to perform impersonation attack because he can’t compromise biometric authentication. But the drawback of this work is, all users need to have fingerprint devices and it may take lot of time to login into the user’s account as the user have to pass three steps of authentication.

Kumar *et al*. [23] proposed a blockchain based framework named “Prodchain” by using lattice based cryptographic processes to minimize the tracing complexity of e-commerce products for the users. This scheme is beneficial for users to trace their desired product in e-commerce cite providing the financial and social sustainability but there is no security to the privacy of the users. Moreover, there is no authentication and registration of sellers, users may get fake information about the products shown in the e-commerce site.

Dou *et al*. [24] suggested multi microgrids which are important in smart grid operations. In this work, multigrid acts as both buyer and supplier for trading of energy. However, the authentication mechanism of multigrid is not addressed in this work. Liu *et al*. [25] suggested a multiparty computation mechanism for securely transferring the shared data. Further, the Paillier encryption method is suggested in this work for sharing of data. However, conditional tracking mechanism is not addressed in this work. Ullah *et al*. [26] introduced the concept of sharing their energy with neighbour’s, i.e., producer and consumers. As a result, they can maximize their energy use without the need for a traditional mediator in the transaction. However, the privacy and authentication of the users are not preserved in this work.

Z. Liu *et al*. [27] proposes a novel privacy-preserving trust management (PPTM) scheme specifically designed for emergency message dissemination in space air ground integrated vehicular networks (SAGIVNs), aiming to address the need for both precise trust management and strong privacy preservation with minimal communication overhead. The work highlights the importance of balancing trust management and privacy preservation in vehicular networks, as both are critical for ensuring the reliability and security of information being shared, especially during emergencies. In order to resolve the contradiction between privacy preservation and trustworthiness in SAGIVNs, three cutting-edge methods are reviewed by Z. Liu *et al*. [28] that analyzes the competing criteria of each. Furthermore, the authors suggest a novel approach that can offer quick decision-making, a method for reputation feedback, traceability, and resistance to Sybil attacks.

Though, the above papers are related to online trading, there is no efficient conditional tracking mechanism for the users/ mediator. Moreover, conditional privacy is achieved in this work. Further, dummy identity is used in this work to preserve the anonymity of the end users. In-addition, due to the minimum certificate and signature verification cost, the work achieves better efficiency.

## 3. System overview

In this part, the system model, the *OTE* attack models, and the bilinear pairing preliminaries are described.

### 3.1 System model

**Fig 1:**
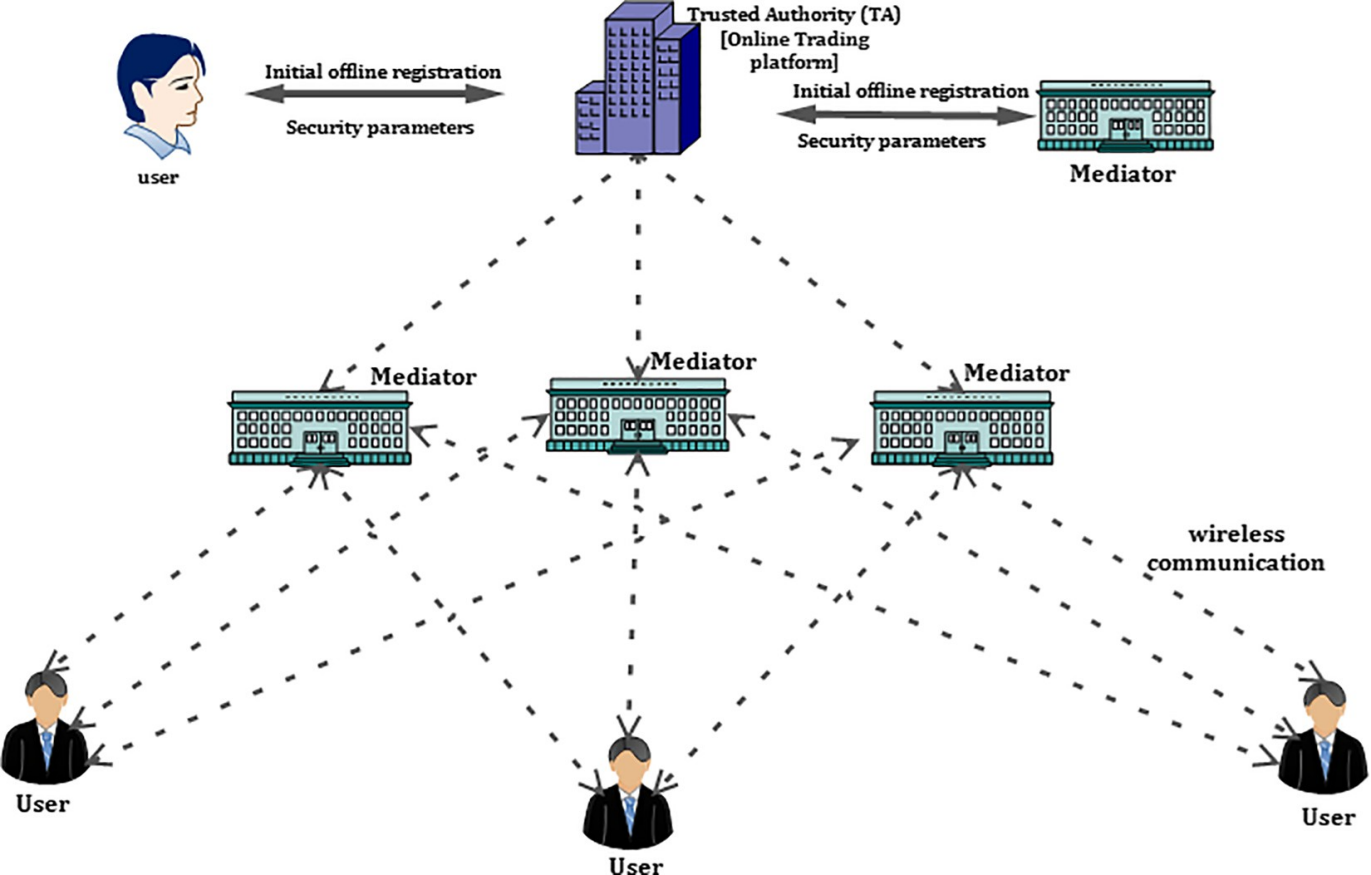
System model of online trading environment.

The system model for the proposed privacy-preserving authentication scheme is demonstrated in Fig 1. It consists of three main entities namely online trading platform (*OTP*), mediators (*M*_*i*_), and users (*U*_*i*_).

- **Online Trading platform (OTP)**: The heart of *OTE* is *OTP*. In *OTE*, *OTP* is known as the trusted party, and it is very difficult for an attacker to compromise *OTP* and it is fully trusted. The *U*_*i*_ and *M*_*i*_ have to register at *OTP* before they started communicating each other. In the proposed scheme, there will be number of *OTP*’s and for each *OTP* there will be a branch in each geographical area for the purpose of offline registration of users and mediators. In Fig 1, the operation of single *OTP* is demonstrated. Moreover, the *OTP* issues all required initial security parameters to all mediators and users at the time of offline registration process. After receiving the demand details from *OTE* users, *OTP* will send this information to *M*_*i*_ in a secure manner.
- **Mediators (*M***_***i***_): Initially, the *M*_*i*_ should register under one specific *OTP* based on their interest. *M*_*i*_ is an intermediate between the seller and buyer (*U*_*i*_).The *M*_*i*_ is connected to *OTP* and *U*_*i*_ in a wireless manner. In this scheme *M*_*i*_ is known to be a semi trusted party. If they disclose any internal data to any attacker or the particular *M*_*i*_ is found to be compromised, then the *OTP* will track the original identity of that *M*_*i*_ and revoke its access within a short time. The *M*_*i*_ will receive demand information of users from *OTP* and based on the existing information it provides the information to the users.
- **User (*U***_***i***_**):** The *U*_*i*_ can communicate with other entities in *OTE* by deploying an online trading device (*OTD*) in their devices. To perform secure and convenient trading, the *OTD*s are deployed to exchange information with *OTP* and *M*_*i*_ by using secret keys which are reserved in it for secure and anonymous communication. A user (*U*_*i*_) can be a seller (*S*_*i*_) or a buyer (*B*_*i*_). Moreover, if he wants to sell any particular goods to others or to buy any goods from others, then he will send his demand information to the *OTP*.

### 3.2 Security attack model in OTE

In our proposed *OTE* platform, there are two types of attackers i.e., internal and external attackers. The internal attackers are compromised *M*_*i*_ *and U*_*i*_ in the *OTE* network. In addition, the internal attacker contains *OTE* secret keys as they are the part of the proposed system. The proposed scheme is mainly concentrated on an external attacker. External attackers are known to be the powerful attackers than internal attackers. Because, the external attacker can perform masquerade attack to enter into *OTE* by acting as an authorized user such as *M*_*i*_ *or U*_*i*_. Different possible circumstances of the attack in *OTE* are discussed below.

- **Fake message attack:** An attacker may send fake information to other entities in *OTE* to perform any specific task. For example, an attacker can send a false message to another *OTE* user as a *M*_*i*_ by stating that he has a perfect deal for sale even though he didn’t have any sale.
- **Impersonation attack:** An attacker can detect the true identity of a *U*_*i*_ or *M*_*i*_ successfully and attacker can use it to enter into *OTE* as an authorized user.
- ***M***_***i***_ **replication attack:** *M*_*i*_ is assumed to be semi trusted component in *OTE*, so it is not provided with more protection against any attack. So, the attacker will try to compromise *M*_*i*_. If any *M*_*i*_ is compromised, the attacker can launch any mischievous attack by entering into *OTE*.
- **Identity revealing attack:** This particular attack is mainly concentrated on user’s privacy. By using this attack, an attacker can collect personal or sensitive information from *OTE* users illegally.
- **Certificate and key duplication attack:** An adversary may use the duplicate certificate and keys of other entities as a proof of authentication to confuse *OTP*.
- **Forgery attack:** An attacker can forge the signature or certificate of the particular information and he can use them for his personal use in the *OTE* network.

### 3.3 Bilinear pairing

Let *G*_*x*_, *G*_*y*_ and *G*_*z*_ denotes the multiplicative cyclic groups of order *p*, here *p* is a large prime number. Assume *g*_*x*_ is the generator of *G*_*x*_, *g*_*y*_ is the generator of *G*_*y*_, and let *φ* be an isomorphism from *G*_*y*_ to *G*_*x*_ such that *φ*(*g*_*y*_) = *g*_*x*_. *e*: *G*_*x*_ *X G*_*y*_→*G*_*z*_ is a bilinear map and below are the bilinear pairing properties.

- Bilinear: $e(g_{x}^{a},g_{y}^{b})={e(g_{x},g_{y})}^{ab}$ for all $g_{x}\in G_{x}\&g_{y}\in G_{y}$ and $\forall a,b\in Z_{q}^{*}$.
- Non-degeneracy: *e*(*g*_*x*_, *g*_*y*_) ≠ 1*G*_*z*_
- Computability: There exists an efficient algorithm to compute the bilinear map *e*: *G*_*x*_×*G*_*y*_→*G*_*z*_.

## 4. Proposed TPAAS scheme

In this section, the *OTE* system’s initialization, user authentication, and mediator authentication processes are explained. Initially, user and mediator should register in the *OTP*’s official website with their credentials i.e., the user/mediator will send a message to *OTP* with their certificate and signature through offline mode. Then the *OTP* will anonymously authenticate the user/mediator in an anonymous manner. Once the authentication is successfully completed, the *OTP* allows the user/mediator to access the resources on the *OTP*’s website. If a user wishes to sell or buy a product, he must send his demand/sale information to *OTP*. The *OTP* will send this demand information to the authenticated mediators in the user’s area. After receiving the demand information from *OTP*, each mediator will use the information already possessed by them to offer best deal for the user.

For instance, if a user wants to buy a product, the information is fed into the *OTP*, and the *OTP* convey the same message to the mediator. The mediators will look for a seller who wants to sell a similar product. If no such sellers are available, the mediator stores the product’s demand information. Moreover, the mediator will use this demand information in the future if he receives any information about the sale of the same specific product from any seller. When the demand information is satisfied, the mediator will send a message to the user (buyer) who requested that specific product. This process is carried out by all network mediators to the required user. After receiving all the messages from the mediators, user will choose one best deal from one of the mediators. In addition, the user will authenticate that specific mediator in an anonymous manner to ensure the message’s integrity. When the user successfully completes the mediator authentication, the user acknowledges the purchase/sale of the product. Similarly, the mediator check for the authenticity of the user. Thus, a mutual anonymous authentication takes place between the user and mediator in a secured manner. Any receiver entity in *OTE* will authenticate and check the legitimacy and integrity of any message before accepting that message from any sender entity. Here, the verification of integrity and legitimacy of any message is done by using bilinear pairing method.

**Fig 2:**
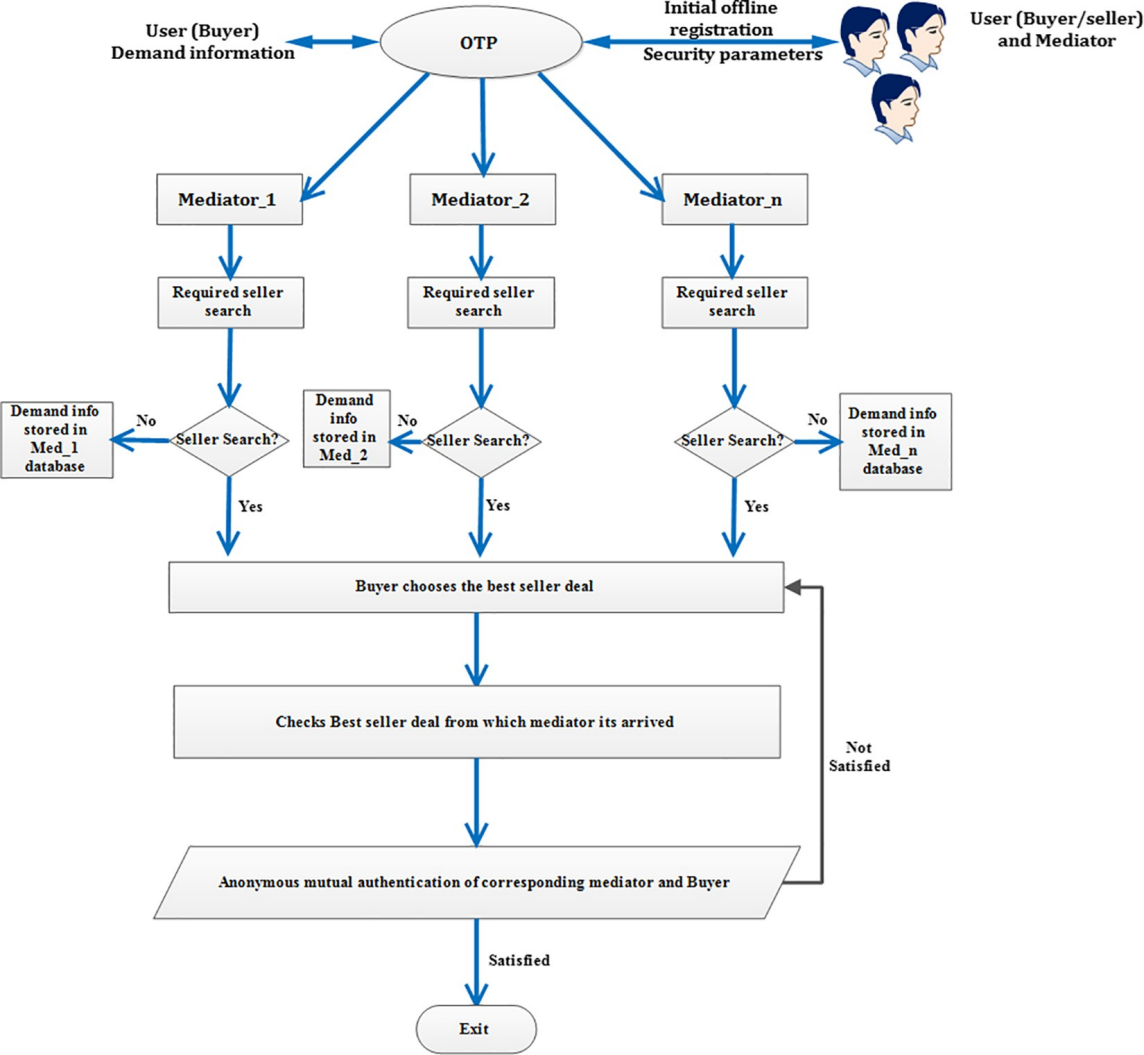
Flowchart for complete flow of information between the Buyer and the OTP through mediator.

**Fig 3:**
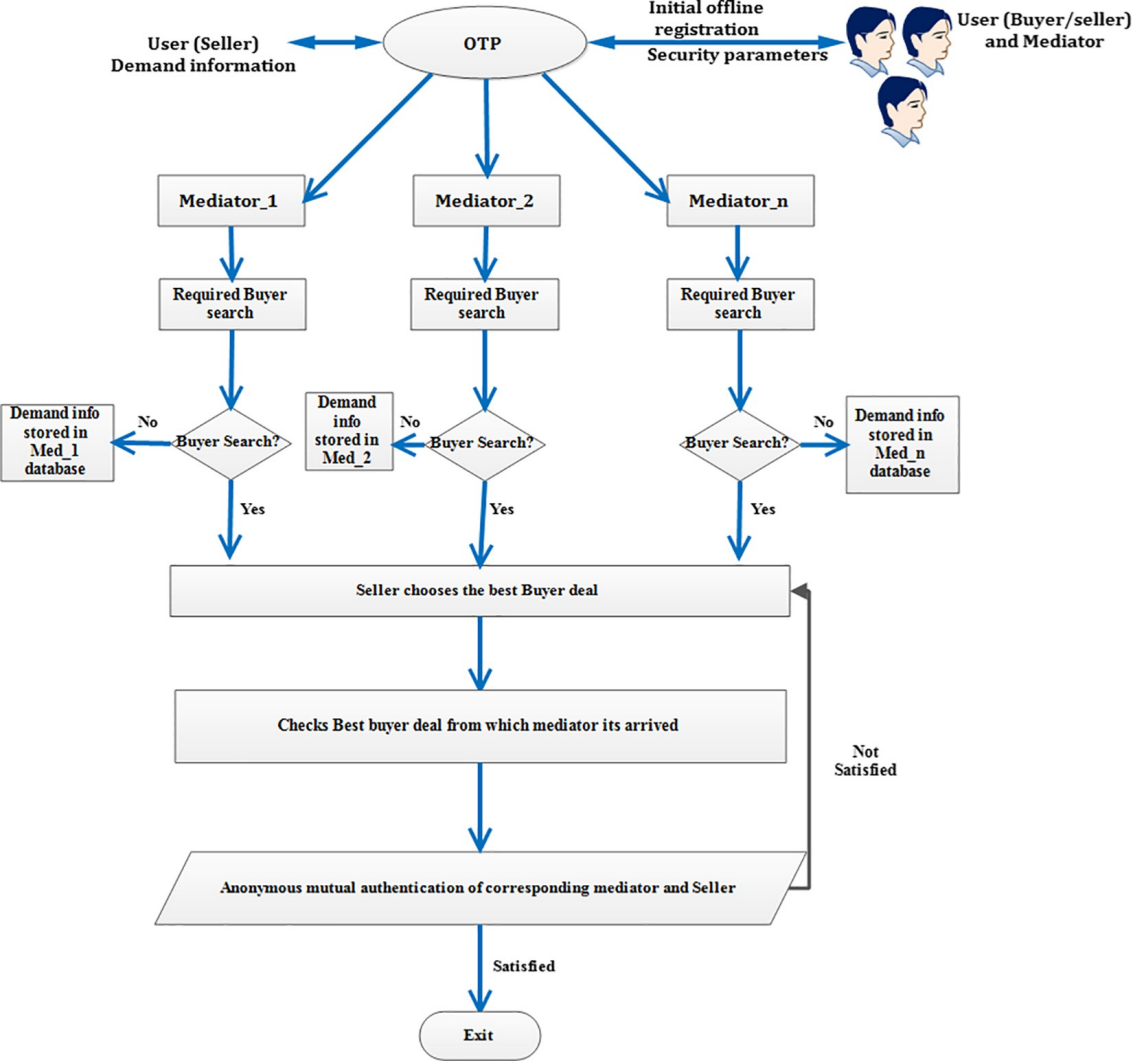
Flowchart for complete flow of information between the seller and the OTP through mediator.

The Fig 2, flowchart shows the complete flow of exchange of information between the buyer and OTP through mediator. Similarly, Fig 3 flowchart shows the complete flow of exchange of information between the seller and OTP through mediator. The explanation for Fig 2 flowchart is as follows. Initially, both the seller/ buyer and mediator should register in OTP using their required credentials and security parameters are issued to them. Further, the buyer demand information is stored in OTP. This demand information is informed to the nearby mediators in an online way. The mediators perform the required seller’s search. Once, the required seller is identified, the information of the seller is conveyed to the buyer by the corresponding mediator. Now, the buyer has all the information of the sellers from different mediators. The buyer decides the best deal among the different sellers. Once the best deal is chosen, the corresponding mediator from which the best deal chosen is identified by the buyer. Thereafter, mutual anonymous authentication takes place between the corresponding buyer and the mediator. If, the mutual anonymous authentication fails, once again the buyer chooses another best deal from another mediator and the process repeats. On the other hand, if authentication is satisfied, then the corresponding mediator deal is accepted and the process completes. The same procedure is to be followed for Fig 3 flowchart.

### 4.1 System initialization

The *OTP* issues system parameters by using bilinear parameters (*G*_*x*_, *G*_*y*_, *G*_*z*_, *p*, *e*) as follows. Initially, the *OTP* selects the random numbers $r,s,t\in Z_{p}^{*}$ as the master secret keys and computes $R_{1}=g_{x}^{r},\;S_{1}=g_{x}^{s}{,\;T}_{1}=g_{x}^{t}$. Then *OTP* selects the secure cryptographic hash function $H:\;{\left\{0,1\right\}}^{*}\to Z_{p}^{*}.$ Finally, the *OTP* issues the *OTE* parameters and broadcast in the open network platform as ${OTE}_{param}=(G_{x},G_{y},G_{z},p,g_{x},g_{y},R_{1},S_{1},T_{1},H,e)$. Table 1 shows the notations used.

**Table 1:** Notations used in the proposed work.

Symbol	Description
*r*, *s*, *t*, *a*_*i*_, *b*_*i*_	**Master secret keys of OTP**
*R*_1_, *S*_1_, *T*_1_, *A*_*i*_, *B*_*i*_, *P*_*i*_	**Public keys of OTP**
*H*: {0,1}*	**Cryptographic hash function**
*URI*_*i*_, *MRI*_*i*_	**User and mediator real identities**
*UDI*_*i*_, *MDI*_*i*_	**User and mediator dummy identities**
*n*_*i*_, *d*_*i*_, *u*, *x*_1_, *x*_2_, *x*_3_	**Random numbers generated by *U*_*i*_**
*α*, *α*_1_, *α*_2_, *α*_3_, *β*, *β*_1_, *β*_2_, *β*_3_	**Public values of *U*_*i*_**
*C*_*i*_, *D*_*i*_	**Tracking parameter for user**
*Ck*	**consumer key for user**
*J*_*i*_, *K*_*i*_	**Internal parameters of *Ck***
*k*_*j*_, *w*_*j*_	**Short time private key of user and mediator**
*N*_*j*_, *μ*	**Short time public key of user and mediator**
$Ak,Ak_{m_{i}}$	**User and mediator acceptor key**
${cert}_{j},{cert}_{m_{i}}$	**Anonymous certificate of user and mediator**
$Message,{Message}_{m_{i}}$	**User and mediator anonymous message**
$sig,{sig}_{m_{i}}$	**Signature for user and mediator**
*Mk*	**Mediator key**
*h*, *f*_1_, *f*_2_, *f*_3_	**Random numbers generated by mediator**
*γ*, *γ*_1_, *γ*_2_, *γ*_3_, *δ*_1_, *δ*_2_, *δ*_3_	**Public values of mediator**
$Ak_{m_{i}}$	**mediator acceptor key**

### 4.2 User (*U*_*i*_) authentication

**Fig 4:**
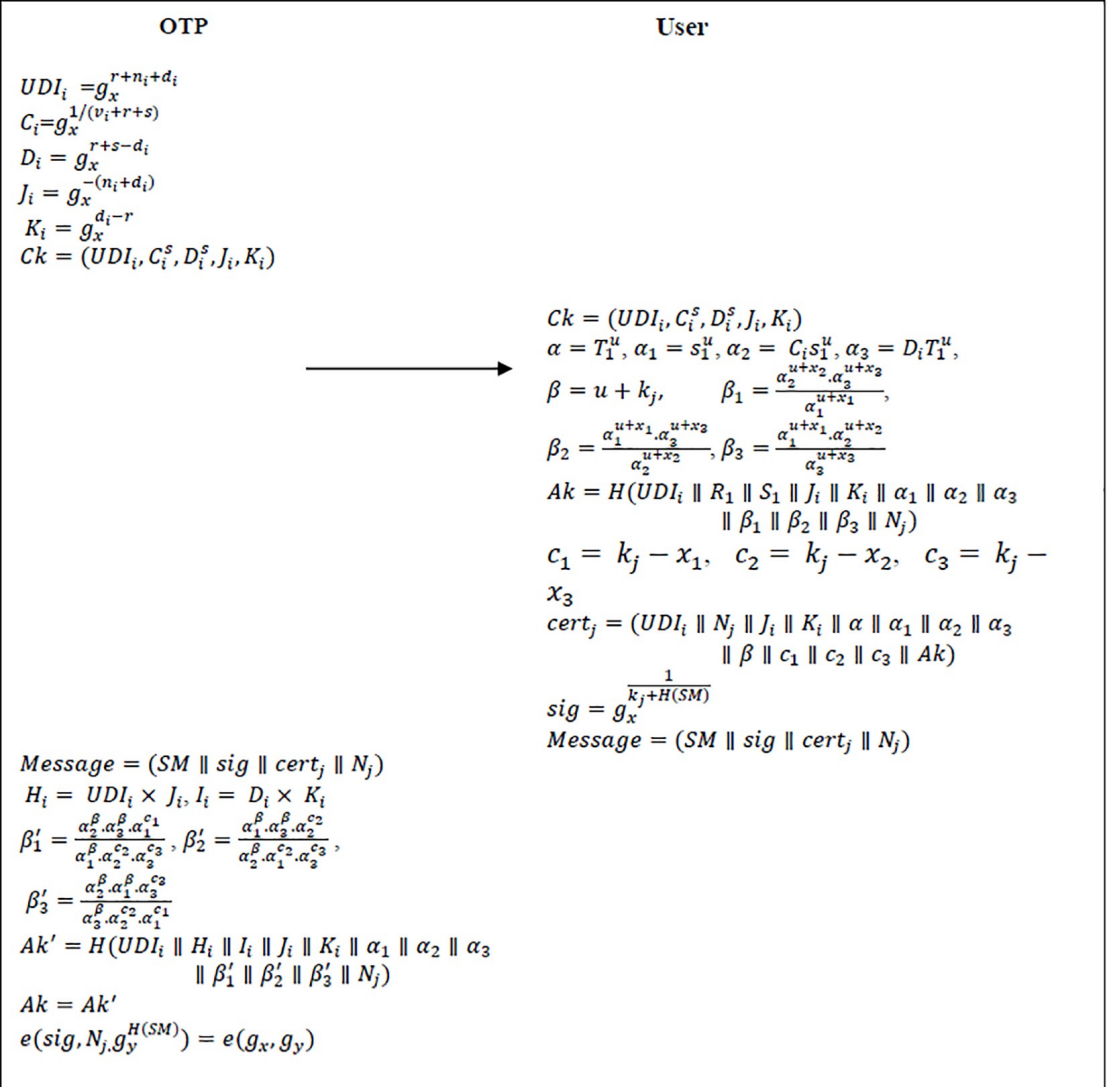
User authentication phase of proposed scheme.

In this proposed scheme, the anonymous *U*_*i*_ authentication process consists of user registration, required keys generation, generation of anonymous certificate, generation of signature, verification of both certificate and signature and conditional tracking. Fig 4 shows the user authentication scheme for proposed scheme.

- **User registration:** To access the *OTP* resources, the user should register at *OTP* in advance in an offline manner. While registration, the user (*U*_*i*_) have to submit the necessary information like credit card details, bank account details, user name, address, username, mail ID, phone number, etc., to the *OTP*. Once the registration process is successfully completed, the user becomes an *OTP* user/entity.
- **Key Generation:** After user registration, the *OTP* will generate necessary secret keys for the user (*U*_*i*_) by using key generation scheme. Initially, the *OTP* generates the user’s (*U*_*i*_) original identity (*URI*_*i*_) and also dummy identity (*UDI*_*i*_). To generate dummy identity *UDI*_*i*_ the *OTP* uses two random numbers *n*_*i*_, $d_{i}\in Z_{p}^{*}$ and then computes ${UDI}_{i}=g_{x}^{r+n_{i}+d_{i}}$. Then the *OTP* maps the original identity with dummy identity by using the tracking list. Moreover, the *OTP* creates dummy identities to all users to check the legitimacy and integrity of the source of information without revealing the actual identity of user to the outside world. Even if the malicious entity finds this dummy identity, they cannot track the original identity of the user. Then the *OTP* selects a random number *v*_*i*_ such that $v_{i}\in Z_{p}^{*}$ and computes $C_{i}=g_{x}^{{1/(v}_{i}+r+s)}$ and $D_{i}=g_{x}^{r+s-d_{i}}$ for tracking the identity of user *U*_*i*_. Further, *OTP* will place the values $\left[{URI}_{i},{UDI}_{i},C_{i}^{s},D_{i}^{s}\right]$ in the tracking list and issues the consumer key (*Ck*) to user as $Ck=({UDI}_{i},C_{i}^{s},D_{i}^{s},J_{i},K_{i})$ and this *Ck* will be kept securely in *OTD*, where $J_{i}=g_{x}^{-(n_{i}+d_{i})}$ and $K_{i}=g_{x}^{d_{i}-r}$. Once the registration and key distribution process is completed, the user *U*_*i*_ can access all the resources of *OTP*’s official website for selling/buying process with the help of its secret keys which are issued by *OTP*.
- **Anonymous certificate Generation:** The user *U*_*i*_ will perform the following steps to generate the required anonymous certificates.

**Step1:** Initially the user will choose random number $k_{j}\in Z_{p}^{*}$ as short time temporary private key and computes the short time public key $N_{j}=g_{y}^{k_{j}}$.

**Step2:** Then the *OTE* user will generate the anonymous one-time certificate *cert*_*j*_ by using their short time public keys *N*_*j*_ as follows:

Initially, the user selects randomly $u,x_{1},x_{2},x_{3}\in Z_{p}^{*}$ and calculates *α*, *α*_1_, *α*_2_, *α*_3_, *β*, *β*_1_, *β*_2_, *β*_3_

Where,

$$\alpha =T_{1}^{u}$$ (1)

$$\alpha_{1}=s_{1}^{u}$$ (2)

$$\alpha_{2}=C_{i}s_{1}^{u}$$ (3)

$$\alpha_{3}=D_{i}T_{1}^{u}$$ (4)

$$\beta ={u+k}_{j}$$ (5)

$$\beta_{1}=\frac{\alpha_{2}^{u+x_{2}}.\alpha_{3}^{u+x_{3}}}{\alpha_{1}^{u+x_{1}}}$$ (6)

$$\beta_{2}=\frac{\alpha_{1}^{u+x_{1}}.\alpha_{3}^{u+x_{3}}}{\alpha_{2}^{u+x_{2}}}$$ (7)

$$\beta_{3}=\frac{\alpha_{1}^{u+x_{1}}.\alpha_{2}^{u+x_{2}}}{\alpha_{3}^{u+x_{3}}}$$ (8)

After computing *α*, *α*_1_, *α*_2_, *α*_3_, *β*, *β*_1_, *β*_2_, *β*_3_, the user will calculate the acceptor key $Ak=H({UDI}_{i}∥R_{1}∥S_{1}∥J_{i}∥K_{i}∥\alpha_{1}∥\alpha_{2}∥\alpha_{3}∥\beta_{1}∥\beta_{2}∥\beta_{3}∥N_{j})$ and then compute the values of *c*_1_, *c*_2_, *c*_3_ as below

$$c_{1}=k_{j}-x_{1}$$ (9)

$$c_{2}=k_{j}-x_{2}$$ (10)

$$c_{3}=k_{j}-x_{3}$$ (11)

Finally, the user will generate the anonymous certificate as ${cert}_{j}=({UDI}_{i}∥N_{j}∥J_{i}∥K_{i}∥D_{i}∥\alpha ∥\alpha_{1}∥\alpha_{2}∥\alpha_{3}∥\beta ∥c_{1}∥c_{2}∥c_{3}∥Ak)$. If user wants to register in other *OTP*, then the user will send an anonymous message to new *OTP* as *Message* = (*SM*∥*sig*∥*cert*_*j*_∥*N*_*j*_). From the received message, the new *OTP* will extract the values of *UDI*_*i*_, *D*_*i*_, *J*_*i*_, *K*_*i*_. Moreover, it will compute *H*_*i*_ = *UDI*_*i*_×*J*_*i*_, *I*_*i*_ = *D*_*i*_×*K*_*i*_ and check whether *H*_*i*_ = *R*_1_ *and I*_*i*_ = *S*_1_. If they are equal, then new OTP accepts the user as an authorized user.

- **Signature Generation:** User will generate the short time signature as $sig=g_{x}^{\frac{1}{k_{j}+H(SM)}}$ to maintain the integrity of the message. Then the user broadcasts the message to other entities as *Message* = (*SM*∥*sig*∥*cert*_*j*_∥*N*_*j*_) by appending original message, certificate, signature and short time public key. Initially, the user will send this message to *OTP* while logging in the *OTP*’s website. The user will place his user name in place of *SM* and the *OTP* will authenticate the user and verify the integrity of the message. Once the verification is completed, the *OTP* will allow the user to access all the resources on the *OTP*’s website i.e., User (*U*_*i*_) authentication by the *OTP*. Similarly, when the user responds to the mediator product request, user will send this *Message* to mediator for the purpose of authentication. Thus, the mediator will authenticate the user i.e., User (*U*_*i*_) authentication by the Mediator (*M*_*i*_). In the above mentioned two cases, the receiver (either the *OTP* or the mediator) will authenticate the user by using *U*_*i*_’s certificate (*Cer*_*j*_) and signature (*Sig*_*j*_) by using the verification process.
- **Verification process:** After receiving the *Message* = (*SM*∥*sig*∥*cert*_*j*_∥*N*_*j*_) from user, the receiver (either the *OTP* or the mediator) will perform below steps to authenticate the user.

**Step 1:** To check the authenticity of the source message, the receiver will calculate $H_{i},I_{i},\beta_{1}^{\prime },\beta_{2}^{\prime },\beta_{3}^{\prime }$ parameters. Where,

$$H_{i}={UDI}_{i}\times J_{i}$$ (12)

$$I_{i}=D_{i}\times K_{i}$$ (13)

$$\beta_{1}^{\prime }=\frac{\alpha_{2}^{\beta }.\alpha_{3}^{\beta }.\alpha_{1}^{c_{1}}}{\alpha_{1}^{\beta }{.\alpha }_{2}^{c_{2}}.\alpha_{3}^{c_{3}}}$$ (14)

$$\beta_{2}^{\prime }=\frac{\alpha_{1}^{\beta }.\alpha_{3}^{\beta }{.\alpha }_{2}^{c_{2}}}{\alpha_{2}^{\beta }{.\alpha }_{1}^{c_{2}}{.\alpha }_{3}^{c_{3}}}$$ (15)

$$\beta_{3}^{\prime }=\frac{\alpha_{2}^{\beta }.\alpha_{1}^{\beta }.\alpha_{3}^{c_{3}}}{\alpha_{3}^{\beta }{.\alpha }_{2}^{c_{2}}{.\alpha }_{1}^{c_{1}}}$$ (16)

**Step 2:** By using above parameters receiver computes $Ak^{\prime }=H({UDI}_{i}∥H_{i}∥I_{i}∥J_{i}∥K_{i}∥\alpha_{1}∥\alpha_{2}∥\alpha_{3}∥\beta_{1}^{\prime }∥\beta_{2}^{\prime }∥\beta_{3}^{\prime }∥N_{j})$ and it verifies whether *Ak* = *Ak*′. If it holds, the receiver will accept the user’s message otherwise it will be discarded. Moreover, the receiver will also check the dummy identity of user by calculating *H*_*i*_ and *I*_*i*_.

**Step 3:** After the completion of acceptor key verification, the receiver will now check the integrity of the message as below.


$$e(sig,N_{j.}g_{y}^{H(SM)})=e(g_{x},g_{y})$$ (17)

If it holds, then the receiver will accept the message otherwise the *Message* will be rejected.

- **Conditional Tracking:** If any conflict occurs or any user is compromised, then the *OTP* can easily detect the real identity of that user by using tracking parameters $C_{i}^{s}$ and $D_{i}^{s}$. The *OTP* will calculate the $\frac{\alpha_{2}^{s}}{\alpha_{1}^{r}},\frac{\alpha_{3}^{s}}{\alpha^{s}}$ to get $C_{i}^{s}$ and $D_{i}^{s}$ with the help of the ${cert}_{j}=({UDI}_{i}∥N_{j}∥J_{i}∥K_{i}∥\alpha ∥\alpha_{1}∥\alpha_{2}∥\alpha_{3}∥\beta ∥c_{1}∥c_{2}∥c_{3}∥Ak)$.

Once $C_{i}^{s}$ and $D_{i}^{s}$ is calculated, the *OTP* will map these parameters to original identity of user by using the tracking list. Moreover, the *OTP* will revoke the privacy of the user and will remove the compromised *U*_*i*_ from *OTE* to avoid further damage.

### 4.3 Mediator (*M*_*i*_) authentication

**Fig 5:**
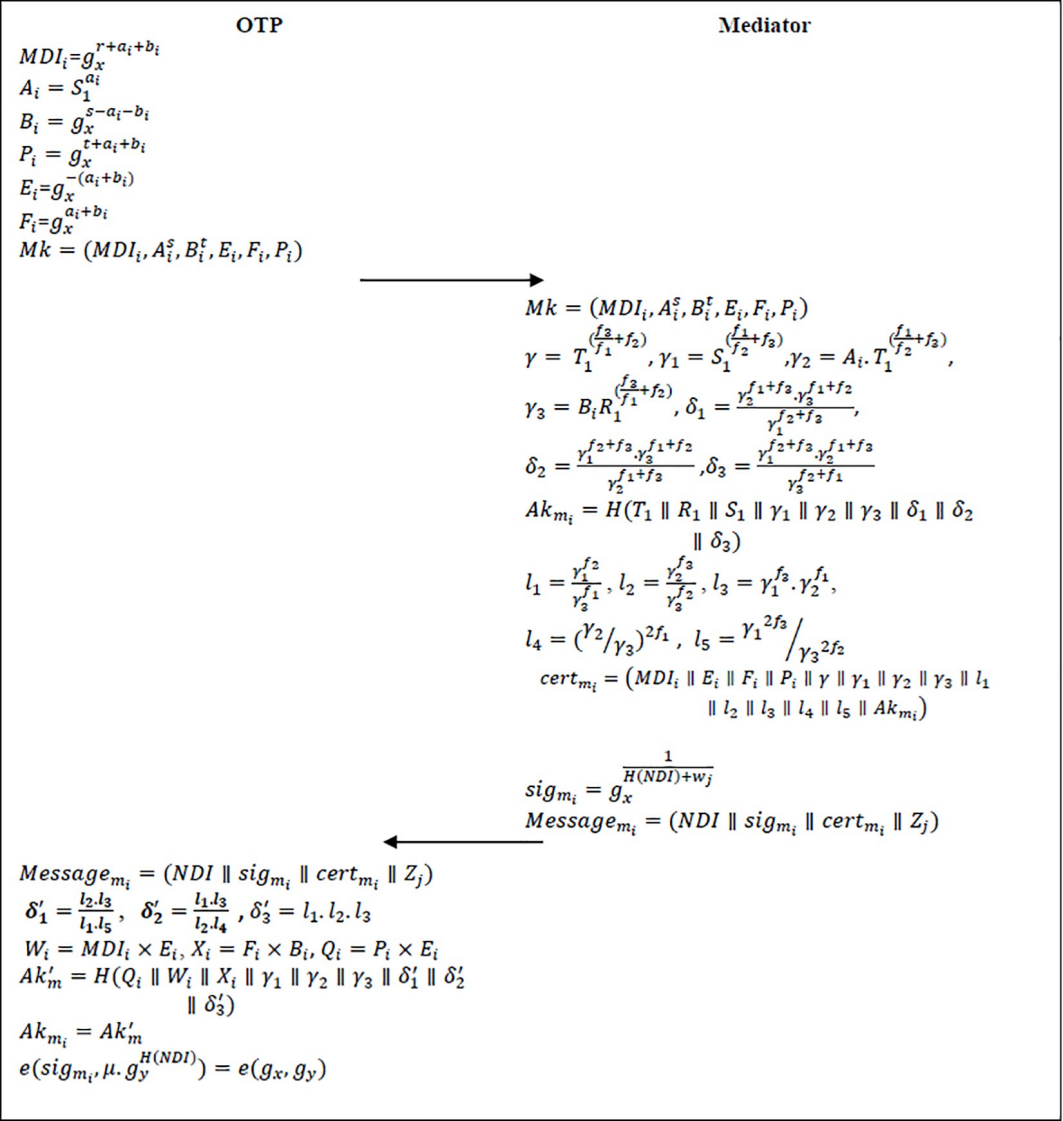
Mediator authentication phase of the proposed scheme.

Once the offline registration is successfully completed, the mediator will log into the official website of *OTP* by sending a message with his signature and certificate to *OTP*. Then, the *OTP* will authenticate the mediator by using the mediator’s certificate and signature to grant him access to the website resources, i.e., Mediator authentication by *OTP*. After receiving the product demand information of user from *OTP*, the mediator will notify the user about the product’s sale. Here, the user must authenticate the mediator in order to avoid receiving messages from a fake mediator or malicious entity, i.e., Mediator authentication by user. For these authentication purposes, the mediator will generate its certificate and signature. The process like mediator registration, key generation, certificate and signature generation, verification and conditional tracking are explained below. Fig 5 shows the mediator authentication phase of the proposed scheme.

- **Mediator Registration:** While offline registration, the mediator is required to submit his full name, address, experience in the e commerce field, username etc., to the *OTP*.
- **Key Generation:** The *OTP* chooses $a_{i},b_{i}\in Z_{p}^{*}$ to calculate *A*_*i*_, *B*_*i*_, *P*_*i*_, where $A_{i}=S_{1}^{a_{i}},B_{i}=g_{x}^{s-a_{i}-b_{i}}$ and $P_{i}=g_{x}^{t+a_{i}+b_{i}}$. Then, the *OTP* generates Mediator real identity (*MRI*_*i*_) and Mediator dummy identity (*MDI*_*i*_). Moreover, the *OTP* keeps the $\left({MRI}_{i},{MDI}_{i},A_{1}^{s},B_{i}^{t}\right)$ in its tracking list, where ${MDI}_{i}=g_{x}^{r+a_{i}+b_{i}}$. In addition, *OTP* sends a mediator key $Mk=({MDI}_{i},A_{i}^{s},B_{i}^{t},E_{i},F_{i},P_{i})$ to every mediator, where $E_{i}=g_{x}^{-(a_{i}+b_{i})}{,F}_{i}=g_{x}^{a_{i}+b_{i}}$.
- **Certificate Generation:** The Mediator (*M*_*i*_) chooses random numbers $h,f_{1},f_{2},f_{3}\in Z_{p}^{*}$

to compute *γ*, *γ*_1_, *γ*_2_, *γ*_3_, *δ*_1_, *δ*_2_, *δ*_3_,where

$$\gamma =T_{1}^{\left(\frac{f_{3}}{f_{1}}+f_{2}\right)}$$ (18)

$$\gamma_{1}=S_{1}^{\left(\frac{f_{1}}{f_{2}}+f_{3}\right)}$$ (19)

$$\gamma_{2}=A_{i}.T_{1}^{\left(\frac{f_{1}}{f_{2}}+f_{3}\right)}$$ (20)

$$\gamma_{3}=B_{i}R_{1}^{\left(\frac{f_{3}}{f_{1}}+f_{2}\right)}$$ (21)

$$\delta_{1}=\frac{\gamma_{2}^{f_{1}+f_{3}}.\gamma_{3}^{f_{1}+f_{2}}}{\gamma_{1}^{f_{2}+f_{3}}}$$ (22)

$$\delta_{2}=\frac{\gamma_{1}^{f_{2}+f_{3}}.\gamma_{3}^{f_{1}+f_{2}}}{\gamma_{2}^{f_{1}+f_{3}}}$$ (23)

$$\delta_{3}=\frac{\gamma_{1}^{f_{2}+f_{3}}.\gamma_{2}^{f_{1}+f_{3}}}{\gamma_{3}^{f_{2}+f_{1}}}$$ (24)

Using the above values, *M*_*i*_ will calculate mediator acceptor key as $Ak_{m_{i}}=H(T_{1}∥R_{1}∥S_{1}∥\gamma_{1}∥\gamma_{2}∥\gamma_{3}∥\delta_{1}∥\delta_{2}∥\delta_{3})$ and *l*_1_, *l*_2_, *l*_3_, *l*_4_, *l*_5_.

Where,

$$l_{1}=\frac{\gamma_{1}^{f_{2}}}{\gamma_{3}^{f_{1}}}$$ (25)

$$l_{2}=\frac{\gamma_{2}^{f_{3}}}{\gamma_{3}^{f_{2}}}$$ (26)

$$l_{3}=\gamma_{1}^{f_{3}}.\gamma_{2}^{f_{1}}$$ (27)

l4=(γ2γ3)2f1 (28)

l5=γ12f3γ32f2 (29)

Then mediator generates ${cert}_{m_{i}}=({MDI}_{i}∥E_{i}∥F_{i}∥P_{i}∥\gamma ∥\gamma_{1}∥\gamma_{2}∥\gamma_{3}∥l_{1}∥l_{2}∥l_{3}∥l_{4}∥l_{5}∥Ak_{m_{i}})$ as its anonymous certificate. Since, the certificate is not having any information about the real identity of the mediator, it is impossible for any adversary to reveal the privacy of the mediator.

- **Signature Generation:** Now the mediator *M*_*i*_ will generate its signature to maintain the integrity of information as follows.

**Step 1:** The mediator selects random number $w_{j}\in Z_{p}^{*}$ as short-time private key and calculates the short time public key as $\mu =g_{y}^{w_{j}}$.

**Step 2:** Now *M*_*i*_ calculates the signature as ${sig}_{m_{i}}=g_{x}^{\frac{1}{H(NDI)+w_{j}}}$ by using values of private keys *w*_*j*_, here *NDI* is nearby deal information that is sent by the mediator to the user. Then, the mediator broadcasts the message to the user by using signature as ${Message}_{m_{i}}=(NDI∥{sig}_{m_{i}}∥{cert}_{m_{i}}∥\mu )$.

- **Verification:** After receiving ${Message}_{m_{i}}$ the receivers (*OTP* or User) should authenticate the mediator as follows. The receiver first computes below values.

$$\delta_{1}^{\prime }=\frac{l_{2}.l_{3}}{l_{1}.l_{5}}$$ (30)

$$\delta_{2}^{\prime }=\frac{l_{1}.l_{3}}{l_{2}.l_{4}}$$ (31)

$$\delta_{3}^{\prime }={l_{1}.l}_{2}.l_{3}$$ (32)

Based on these parameters, the mediator calculates the acceptor key as $Ak_{m_{i}}^{\prime }=H(Q_{i}∥W_{i}∥X_{i}∥\gamma_{1}∥\gamma_{2}∥\gamma_{3}∥\delta_{1}^{\prime }∥\delta_{2}^{\prime }∥\delta_{3}^{\prime })$ where, *W*_*i*_ = *MDI*_*i*_×*E*_*i*_, *X*_*i*_ = *F*_*i*_×*B*_*i*_ and *Q*_*i*_ = *P*_*i*_×*E*_*i*_ and checks whether $Ak_{m_{i}}=Ak_{m_{i}}^{\prime }$. If it holds, the receiver accepts the mediator as authorized mediator otherwise it will reject the message of the mediator.

Once the certificate is verified, the receiver will now verifies the integrity of the *NDI* by checking the following equation.


$$e({sig}_{m_{i}},\mu .g_{y}^{H(NDI)})=e(g_{x},g_{y})$$ (33)

If it satisfies, then the receiver (*OTP* or User) will accepts the ${Message}_{m_{i}}$ otherwise it will be rejected.

- **Conditional Tracking:** If there is any conflict or any mediator is compromised, then the *OTP* will track the real identity of that specific mediator in a short time to revoke its privacy. By using certificate ${cert}_{m_{i}}=({MDI}_{i}∥E_{i}∥F_{i}∥P_{i}∥\gamma ∥\gamma_{1}∥\gamma_{2}∥\gamma_{3}∥l_{1}∥l_{2}∥l_{3}∥l_{4}∥l_{5}∥Ak_{m_{i}}∥\mu )$, the *OTP* will compute the values of $A_{i}^{s}$ and $B_{i}^{t}$. Once these values are computed, the *OTP* will map them to the real identity *MRI*_*i*_ of mediator by using the tracking list. The *OTP* calculates $\frac{\gamma_{2}^{s}}{\gamma_{1}^{t}},\frac{\gamma_{3}^{t}}{\gamma^{r}}$ to find $A_{i}^{s}$ and $B_{i}^{t}$.

Now, calculating these values, the *OTP* can easily find the real identity of the mediator. Moreover, the *OTP* will withdraw the privacy of the particular mediator and remove it from the *OTE* system to avoid further damage.

## 5. Security analysis

The source authentication, message integrity, identity privacy preserving and conditional privacy preserving of our proposed anonymous authentication scheme in *OTE* system are explained and analyzed in this section. In our proposed scheme, the signature ‘*sig*’ and certificate ‘*cert*_*j*_’ are required parameters to provide protection against various types of attacks such as impersonate, masquerade, inject, and key replication attacks. In our scheme, it is impossible for an external attacker (*EA*) to calculate a valid certificate and signature using the certificate and signature of another authorised entity, because the consumer key (*Ck*) or mediator key (*Mk*) of the entity that is given by *OTP* in offline is kept securely in *OTD* by the authenticated user or mediator. Hence, it is impossible for an intruder to perform key replication attacks and to send fake messages in the *OTE* system. Moreover, in order to perform an impersonation attack, the intruder must crack the short time private key of the specific user and also find the *Ck or Mk* of the user or mediator given by *OTP* in an offline manner. So, it is impossible to get effected by the impersonation attack. In addition, the *EA* cannot compromise the registration step which is done in the offline mode at the *OTP*. Hence, our *OTE* system is completely secure against impersonation attacks. *OTE* system’s defense procedure against various attacks and threats is explained below.

### 5.1 Defense against impersonation attack

To execute an impersonation attack by pretending to be an authorized user or an authorized mediator, the intruder have to find the secret parameter of the authorized user or mediator i.e., the value of *C*_*i*_ *and D*_*i*_ of the user and *A*_*i*_ *and B*_*i*_ of the mediator. In order to find values of *C*_*i*_ *and D*_*i*_, attacker must find the values of *α*_2_ *and α*_3_ in the user’s certificate ${cert}_{j}=({UDI}_{i}∥N_{j}∥J_{i}∥K_{i}∥\alpha ∥\alpha_{1}∥\alpha_{2}∥\alpha_{3}∥\beta ∥c_{1}∥c_{2}∥c_{3}∥Ak)$. In addition, to find the values of *A*_*i*_ *and B*_*i*_, attacker must find the values of *γ*_2_ and *γ*_3_ in the mediator’s certificate ${cert}_{m_{i}}=({MDI}_{i}∥E_{i}∥F_{i}∥P_{i}∥\gamma ∥\gamma_{1}∥\gamma_{2}∥\gamma_{3}∥l_{1}∥l_{2}∥l_{3}∥l_{4}∥l_{5}∥Ak_{m_{i}}∥\mu )$. The values of *α*_2_ and *α*_3_ are computed as $\alpha_{2}=C_{i}s_{1}^{u}$ and $\alpha_{3}=D_{i}T_{1}^{u}$. Here, ‘*u’* value is selected randomly by the user *U*_*i*_ hence the *α*_2_ *and α*_3_ values are also random. However, the calculation of *α*_2_ and *α*_3_ values involves Elliptic Curve Discrete Logarithm problem (ECDLP). So, there is $O[p^{\frac{1}{2}+o(1)}\;log\;log\emptyset ]$ computational complexity in finding the values of *C*_*i*_ *and D*_*i*_, where ‘∅’ represents the number of users. Moreover, there is a complexity of *O*(2^*m*^−1) in finding the value of ‘*u*’ from set of ‘*m*’ short time random keys. Similarly, in the case of pretending to be a mediator and to find the values of *A*_*i*_ *and B*_*i*_, the values of *γ*_2_ *and γ*_3_ are computed as *γ*_2_ = *A*_*i*_. $T_{1}^{\left(\frac{f_{1}}{f_{2}}+f_{3}\right)}$ and $\gamma_{3}=B_{i}R_{1}^{\left(\frac{f_{3}}{f_{1}}+f_{2}\right)}$. Here, the values of *f*_1_, *f*_2_ *and f*_3_ are selected randomly by the mediator *M*_*i*_ hence the *γ*_2_ *and γ*_3_ values are also random and they computed based on ECDLP with complexity of $\left[q^{\frac{1}{2}+o(1)}log\;log\Delta \right]$, where ‘Δ’ represents the number of mediators. Moreover, there is a complexity of *O*(2^*x*^−1) in finding the values of ‘*f*_1_, *f*_2_ *and f*_3_’ from set of ‘*x*’ short time random keys. Therefore, it is impossible for an *EA* to find the values of *C*_*i*_, *D*_*i*_, *A*_*i*_, *B*_*i*_, u, *f*_1_, *f*_2_ *and f*_3_ to compromise the certificate authentication step and to perform impersonation attack (either by pretending to be an authorized user or mediator) in a stipulated time. Hence, our authentication scheme in *OTE* system can withstand against the impersonation attack.

### 5.2 Defense against bogus message attack

An external attacker ‘*EA*’ has to find the values of *UDI*_*i*_, *J*_*i*_, *D*_*i*_, *K*_*i*_ to send bogus message to *OTE* user. Here, *H*_*i*_ = *UDI*_*i*_×*J*_*i*_ = *R*_1_ and *I*_*i*_ = *D*_*i*_×*K*_*i*_ = *S*_1_. Moreover, the values ${UDI}_{i}=g_{x}^{r+n_{i}+d_{i}},J_{i}=g_{x}^{-(n_{i}+d_{i})},D_{i}=g_{x}^{r+s-d_{i}}$ and $K_{i}=g_{x}^{d_{i}-r}$ are generated for each user directly during the initial offline registration by *OTP*. It is not possible for an *EA* to find the values of *n*_*i*_, *d*_*i*_, *r* and *s* from *UDI*_*i*_, *J*_*i*_, *D*_*i*_ and *K*_*i*_ because of ECDLP. Since, *n*_*i*_ and *d*_*i*_ values are randomly selected by *OTP* and the values *r*, *s* are master keys which are known only to *OTP*. To find the values of *or s*, there is a complexity of $O[p^{\frac{1}{2}+o(1)}log\Theta ]$ where ‘Θ’ represents number of users registered in the *OTP*. To find the values of *n*_*i*_ or *d*_*i*_ there is a complexity of *O*[2^Θ^−1]. Therefore, the total complexity of finding the values of *n*_*i*_, *d*_*i*_, *r* and *s* to compute *UDI*_*i*_×*J*_*i*_ = *R*_1_ and *D*_*i*_×*K*_*i*_ = *S*_1_ are $O([p^{\frac{1}{2}+o(1)}log\Theta ].{\left[2^{\Theta }-1\right]}^{2})$ and $O({\left[p^{\frac{1}{2}+o(1)}log\Theta \right]}^{2}.[2^{\Theta }-1])$. So, it is difficult to perform bogus message attack. Therefore, our authentication scheme in *OTE* system can withstand against bogus message attack.

### 5.3 Defense against message modification attack

In our proposed scheme, every user *U*_*i*_ broadcast his message as "*Message* = (*SM*∥*sig*∥*cert*_*j*_∥*N*_*j*_)". External attackers attempt to inject modifications into this message, such as changing the content of the broadcasted message before it reaches the receiver, while it is transmitting over wireless medium. But in our scheme, to preserve message integrity, user’s signature is generated on a message ‘*SM*’ as $sig=g_{x}^{\frac{1}{k_{j}+H(SM)}}$ where ‘*k*_*j*_’ is the user’s short time private key. Since its value is only known to the user, no other entity can generate the same signature. So, *EA* needs to find the short time private key to forge the signature. However, the value of ‘*k*_*j*_’ changes periodically, so even if *EA* finds the value of short time private key, the ‘*EA*’ cannot follow the subsequent communication related to *U*_*i*_. Similarly, mediator *M*_*i*_ signature on the message ‘*NDI*’ is given by ${sig}_{m_{i}}=g_{x}^{\frac{1}{H(NDI)+w_{j}}}$. Since, the value of ‘*w*_*j*_’ is short time private key which is known only to particular mediator, no other entity can forge it’s signature. Moreover, the users and mediator certificates are generated by using *Ck* and *Mk* values which are given to them securely in an offline manner. So, without knowing the values of *Ck*, *Mk* and short time private keys, *EA* cannot forge the anonymous certificates and signatures.

### 5.4 Conditional privacy preserving

In our proposed scheme, each user and mediator will have an anonymous certificate and signature to hide their real identity. But, if any dispute happens or the user/mediator is compromised, then the *OTP* can trace the original identity of the user or mediator from its anonymous certificate. If the user is compromised, then the *OTP* calculates $\frac{\alpha_{2}^{s}}{\alpha_{1}^{r}},\frac{\alpha_{3}^{s}}{\alpha^{s}}$ to get the values of $C_{i}^{s}$ and $D_{i}^{s}$ from the tracking list. Similarly, if the mediator is compromised, then the *OTP* calculates $\frac{\gamma_{2}^{s}}{\gamma_{1}^{t}},\frac{\gamma_{3}^{t}}{\gamma^{r}}$ to get the value of $A_{i}^{s}$ and $B_{i}^{t}$ from the tracking list. Once if $C_{i}^{s}$ and $D_{i}^{s}$ are traced by the *OTP*, then the fake user will be revoked from the network. Similarly, if $A_{i}^{s}$ and $B_{i}^{t}$ are traced by the *OTP*,then the fake mediator will be revoked from the *OTE*.

### 5.5 Defense against the non repudiation attack

In the proposed scheme, the user (buyer/seller) cannot repudiate after receiving the information from the mediator or sending the demand request to the *OTP*. During receiving the information from the mediator, the authenticity of the user is checked by the mediator using anonymous certificate and signature verification. So, repudiation of the user is not acceptable. Moreover, while sending the demand request to *OTP* by the user, the request can be accepted only, if the user is authenticated. Since, the user gives his credentials and registered in offline to the *OTP*. Once after sending the demand request, the user cannot repudiate.

### 5.6. Anonymity and privacy preservation

In our scheme, users and mediators attach a valid signature and certificate to their messages, so it is computationally hard to trace the actual identity of the user or mediator who signed the message. Moreover, the certificate and signature are computed using dummy identity and short time private keys which are changed periodically. As a result, the *EA* will get zero knowledge about the signer of that message and even if these dummy identities are revealed, they will give zero knowledge about the real identity of the user or mediator. Thus, in our proposed scheme, the anonymity and privacy of the user and mediator are preserved.

### 5.7. Unlinkability

Certificate and signature are generated using temporary private keys. These are short life keys and randomly changeable in a short span of time. So, during information exchange, these short life temporary private keys are used for certificate and signature generation. So, once the information is successfully exchanged and the certificate and signature are validated, the validity of these private keys are expired and a new randomly generated key is to be used for next transaction. So, there is a complete unlinkability during the information exchange.

### 5.8 Defense against man in the middle attack

In a man-in-the-middle attack, the *EA* attempt to modify the messages sent between the user and the mediator. But in our scheme, every *OTE* entity (user/mediator) should first login into the *OTP*’s official website. Only after successful registration, they can access the resources in *OTP*’s website. Therefore, when the user and mediator log into the website, *OTP* will authenticate them using their anonymous certificate and signature, which are computed using the consumer key (*Ck*) and mediator key (*Mk*). But these *Ck and Mk* are issued by *OTP* to the user and mediator during their initial offline registration. So, it is difficult for an intruder to find *Ck* and *Mk* and compute these anonymous certificate and signature. As a result, intruder cannot enter into the *OTE* network without getting authenticated by *OTP*. So, the intruder cannot change the information being transferred between the user to mediator or vice versa. Even-though, if the intruder enter into the network, he cannot change the content of the message, since every entity will attach a valid signature to its message which is calculated by using short time private keys. So, if an intruder wish to modify the content, he needs to compute a valid signature by using short time private keys which are only known to the legitimate users. So, our proposed scheme can withstand against the man in the middle attack.

### 5.9 Defense against the Sybil attack

In this attack, *EA* use multiple identities and send the same message to *OTE* users to make them believe that the message is true as it is coming from different entities. But in our scheme, if *EA* wants to send a single fake message, he has to find the values of *r*, *n*_*i*_ and *d*_*i*_, where dummy identity is calculated by ${UDI}_{i}=g_{x}^{r+n_{i}+d_{i}}$. But, the ‘*r*’ value is a private key which is only known to *OTP* and the values of *n*_*i*_ *and d*_*i*_ are also chosen by *OTP* randomly. So, it is impossible for an *EA* to create multiple identities without knowing *r*, *n*_*i*_ and *d*_*i*_ values. So, in our proposed scheme *EA* cannot send any fake messages to other users by creating multiple identities. Therefore, our scheme can withstand against the Sybil attack.

## 6. Formal security analysis

Formal security is performed using Burrows, Abadi, and Needham (BAN) logic. To ensure mutual authentication between the *U*_*i*_ and OTP, the following goals are defined.


$$G1:\;U_{i}|\equiv U_{i}\overset{Message}{↔}OTP$$ (34)

$$G2:\;U_{i}|\equiv OTP|\equiv U_{i}\overset{Message}{↔}OTP$$ (35)

$$G3:\;OTP|\equiv U_{i}\overset{Message}{↔}OTP$$ (36)

$$G4:\;OTP|\equiv U_{i}|\equiv U_{i}\overset{Message}{↔}OTP$$ (37)

The following idealization is performed for message transformation $U_{i}\overset{Message}{↔}OTP$ as follows.


$$M_{1}:\;OTP\to U_{i}:\;\{{UDI}_{i}\}$$ (38)

$$M_{2}:\;OTP\to U_{i}:\;\{J_{i},K_{i},D_{i}\}$$ (39)

$$M_{3}:\;U_{i}\to OTP:\;\{{Cert}_{j}\}$$ (40)

$$M_{4}:\;U_{i}\to OTP:\;\{{Cert}_{j},N_{j}\}$$ (41)

The security assumptions are made based on the mutual authentication:

$$A_{1}:\;U_{i}|\equiv \#{UDI}_{i}$$ (42)

$$A_{2}:\;U_{i}|\equiv OTP=>{UDI}_{i}$$ (43)

$$A_{3}:\;OTP|\equiv \#(r,s,n_{i},d_{i})$$ (44)

$$A_{4}:\;OTP|\equiv U_{i}=>M_{2}$$ (45)

$$A_{5}:\;OTP|\equiv U_{i}\#{Cert}_{i}$$ (46)

$$A_{6}:\;OTP|\equiv U_{i}=>M_{3}$$ (47)

$$A_{7}:\;OTP|\equiv U_{i}\#(N_{j},sig)$$ (48)

$$A_{8}:\;OTP|\equiv U_{i}=>M_{4}$$ (49)

The mutual anonymous authentication between *U*_*i*_ and *OTP* with the help of the rules mentioned in [29] and based on above assumptions.

From *M*_1_

$$S1:\;U_{i}⊲\{{UDI}_{i}\}$$ (50)

$$S2:\;U_{i}|\equiv OTP|∼{UDI}_{i}\;from\;(S1,A1)$$ (51)

$$S3:\;U_{i}|\equiv OTP|\equiv {UDI}_{i}\;is\;obtained.$$ (52)

Moreover,

$$S4:\;U_{i}|\equiv {UDI}_{i}$$ (53)

From *M*_2_

$$S5:\;U_{i}⊲\{J_{i},K_{i},D_{i}\},$$ (54)

derives

$$S6:\;U_{i}|\equiv OTP|∼(J_{i},K_{i},D_{i})$$ (55)

from *S*5, since $J_{i}=g_{x}^{-(n_{i}+d_{i})},\;K_{i}=g_{x}^{d_{i}-r}$ and $D_{i}=g_{x}^{r+s-d_{i}}$ based on *A*_3_.

$$S7:\;U_{i}|\equiv \#(J_{i},K_{i},D_{i})$$ (56)

$$S8:\;U_{i}|\equiv OTP|\equiv (J_{i},K_{i},D_{i})$$ (57)

from *S*6 and *S*7. Using *A*_4_ and *S*8;

$$S9:\;U_{i}|\equiv (J_{i},K_{i},D_{i})$$ 58

From *M*_3_

$$S10:\;OTP⊲\{{Cert}_{j}\}$$ (59)

$$S11:\;OTP|\equiv U_{i}|∼({Cert}_{j})=(c^{\prime },Ak)=(\beta_{1}^{\prime },\beta_{2}^{\prime },\beta_{3}^{\prime })$$ (60)

$$S12:\;OTP|\equiv \#{Cert}_{j}={\langle Ack\rangle }_{c^{\prime }}={\langle {Cert}_{j}\rangle }_{Ack}$$ (61)

based on *A*_5_. From *S*11 and *S*12;

$$S13:\;OTP|\equiv U_{i}|\equiv {Cert}_{j}$$ (62)

$$S14:\;OTP|\equiv {Cert}_{j}\;based\;on\;A_{6}.$$ (63)

From *M*_4_

$$S15:\;OTP⊲\{sig,N_{j}\}$$ (64)

$$S16:\;OTP|\equiv U_{i}|∼(sig,N_{j})=e(sig,N_{j.}g_{y}^{H(SM)})=e(g_{x},g_{y})$$ (65)

$$S17:\;OTP|\equiv \#(sig,N_{j})\;based\;on\;A_{7}$$ (66)

From *S*16 and *S*17;

$$S18:\;OTP|\equiv U_{i}|\equiv (sig,N_{j})$$ (67)

$$S19:\;OTP|\equiv (sig,N_{j})$$ (68)

$$S20:\;OTP|\equiv {\langle sig\rangle }_{N_{j}}$$ (69)

Based on *S*14 and *S*20, the above desired goals are achieved. The same procedure is repeated for mediator authentication.

## 7. Performance analysis

This section analyses the proposed system’s performance and compares it to other existing systems in terms of computational complexity and communication complexity.

### 7.1 Computational complexity

**Fig 6:**
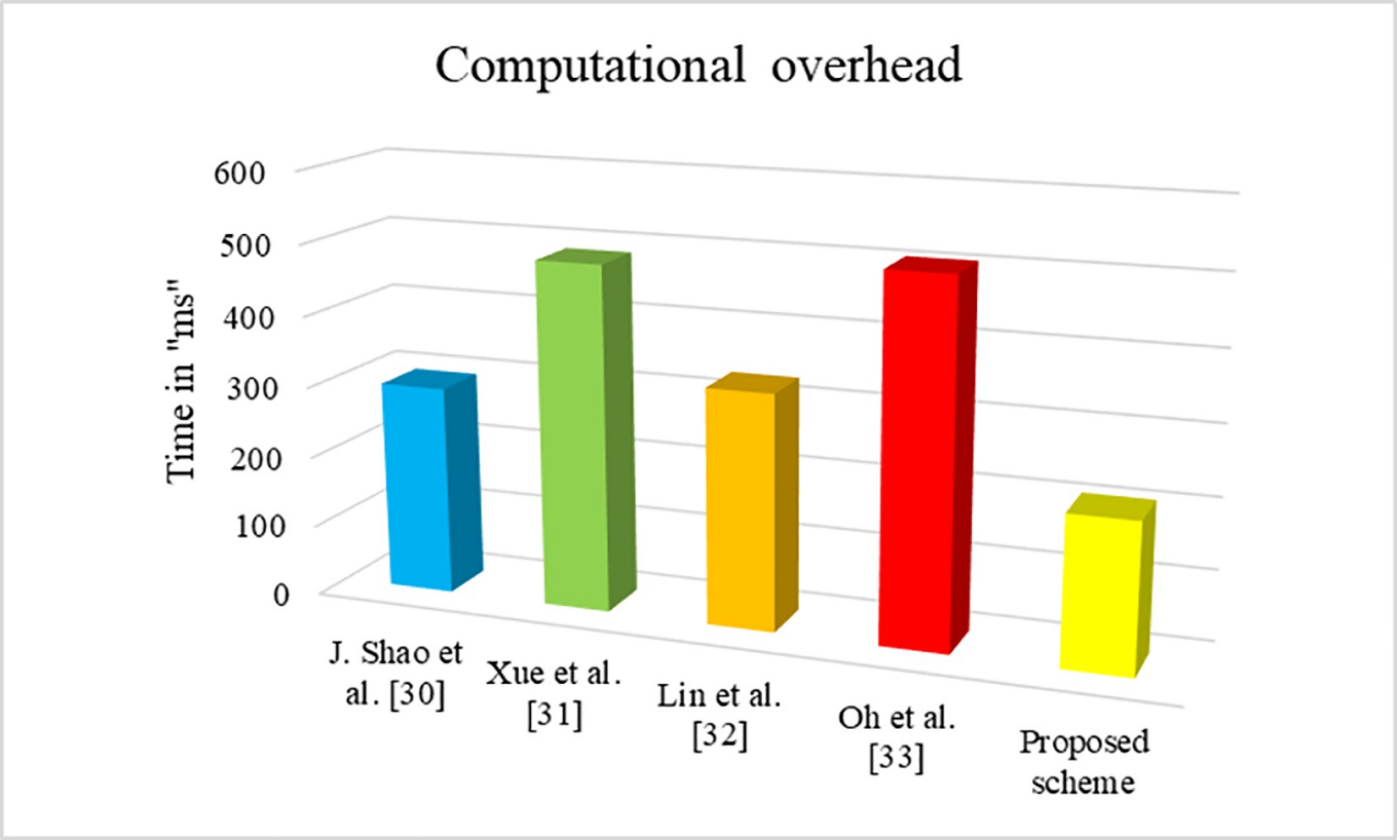
Computational cost for certificate and signature verification.

The computational cost is the amount of time required to verify the certificate and signature. The computational cost of our proposed scheme is compared with different existing schemes such as J. Shao *et al*. [30], Xue *et al*. [31], Lin *et al*. [32], and Oh *et al*. [33]. Several operations such as pairing operation, hash function, one point multiplication and exponential functions are involved during the calculation of computation cost. The time required for performing the pairing operation, hash function, multiplication operation and exponential function are represented as *T*_*p*_, *T*_*h*_, *T*_*m*_ and *T*_*ex*_. In order to perform the computational cost, the proposed scheme is implemented using Ubuntu 22.04, AMD Ryzen 7, 5800U with Radeon Graphics, 1.9 GHz processor, with 16 GB RAM with Cygwin 1.7.35–15 software platform with gcc version 4.9.2 [34]. The bilinear pairing operation has the value of 25.342*ms*. Similarly, the value for hashing operation is 0.2632*ms*, one point addition is 0.0031*ms*, one point multiplication operation is 0.0217*ms*, and exponential operation is 23.242*ms* respectively. The results are calculated for 1000 simulations and finally the average of the results are considered for validation. Here ‘ms’ represents milliseconds. Table 2 shows the computational cost for different schemes. Fig 6 shows the graphical representation of verification time for different schemes based on the received message. The Fig 6 clearly includes the proposed scheme consumes minimum computational time, when compared to the relevant existing schemes.

**Table 2:** Computational cost of various schemes.

Schemes	For single signature and certificate verification	For ‘n’ signature and certificate verification
J. Shao et al. [30]	$3T_{p}+2T_{ex}+2T_{h}$	$(2+n)T_{p}+2nT_{ex}+2nT_{h}$
Xue et al. [31]	$8T_{p}+19T_{ex}+16T_{m}+2T_{a}+7T_{h}$	$8nT_{p}+19nT_{ex}+16nT_{m}+(n+1)T_{a}+7nT_{h}$
Lin et al. [32]	$5T_{p}+13T_{ex}+9T_{m}+2T_{a}+7T_{h}$	$5nT_{p}+13nT_{ex}+9nT_{m}+(n+1)T_{a}+7nT_{h}$
Oh et al. [33]	$2T_{p}+21T_{ex}+5T_{m}+28T_{h}$	$(n+1)T_{p}+21nT_{ex}+5nT_{m}+28nT_{h}$
Proposed scheme	$4T_{a}+9T_{ex}+2T_{m}+T_{h}$	$4nT_{a}+9nT_{ex}+(n+1)T_{m}+nT_{h}$

### 7.2 Communication cost

Communication cost is the number of bits required for the transformation of information between the end users. Table 3 shows the number of messages used for information exchange and communication cost for various schemes. In our proposed scheme, the number of bits assumed for the message used by the user and mediator is *SM* = *NDI* = 160 bits, the signature bits of the *U*_*i*_ and *M*_*i*_ is $sig={sig}_{m_{i}}$ = 160 bits, the certificate bits of the *U*_*i*_ and *M*_*i*_ is ${cert}_{j}={cert}_{m_{i}}$ = 160 bits, *U*_*i*_ and *M*_*i*_ uses the public keys as *N*_*j*_ = *D*_*i*_ = 320 bits [35]. Moreover, in our proposed scheme, two messages are used for the authentication of *U*_*i*_ and *M*_*i*_ and the transformation of messages takes place. The entire messages (information) of user and mediator are represented as *Message* = (*SM*∥*sig*∥*cert*_*j*_∥*N*_*j*_) and ${Message}_{m_{i}}=(NDI∥{sig}_{m_{i}}∥{cert}_{m_{i}}∥Z_{j})$. Both the information consumes *Message* = (160+160+160+320)=800 bits and ${Message}_{m_{i}}$ = (160+160+160+320)=800 bits. So, totally 1600 bits are used as the communication cost for communication in our proposed scheme.

**Table 3:** Communication cost of various schemes.

Scheme	Communication Cost for single user (bits)	Total Communication Cost for *n* users (bits)
J. Shao *et al*. [30]	2912	2912*n*
Xue *et al*. [31]	2080	2080*n*
Lin *et al*. [32]	2240	2240*n*
Oh *et al*. [33]	1760	1760*n*
Proposed scheme	1600	1600*n*

**Fig 7:**
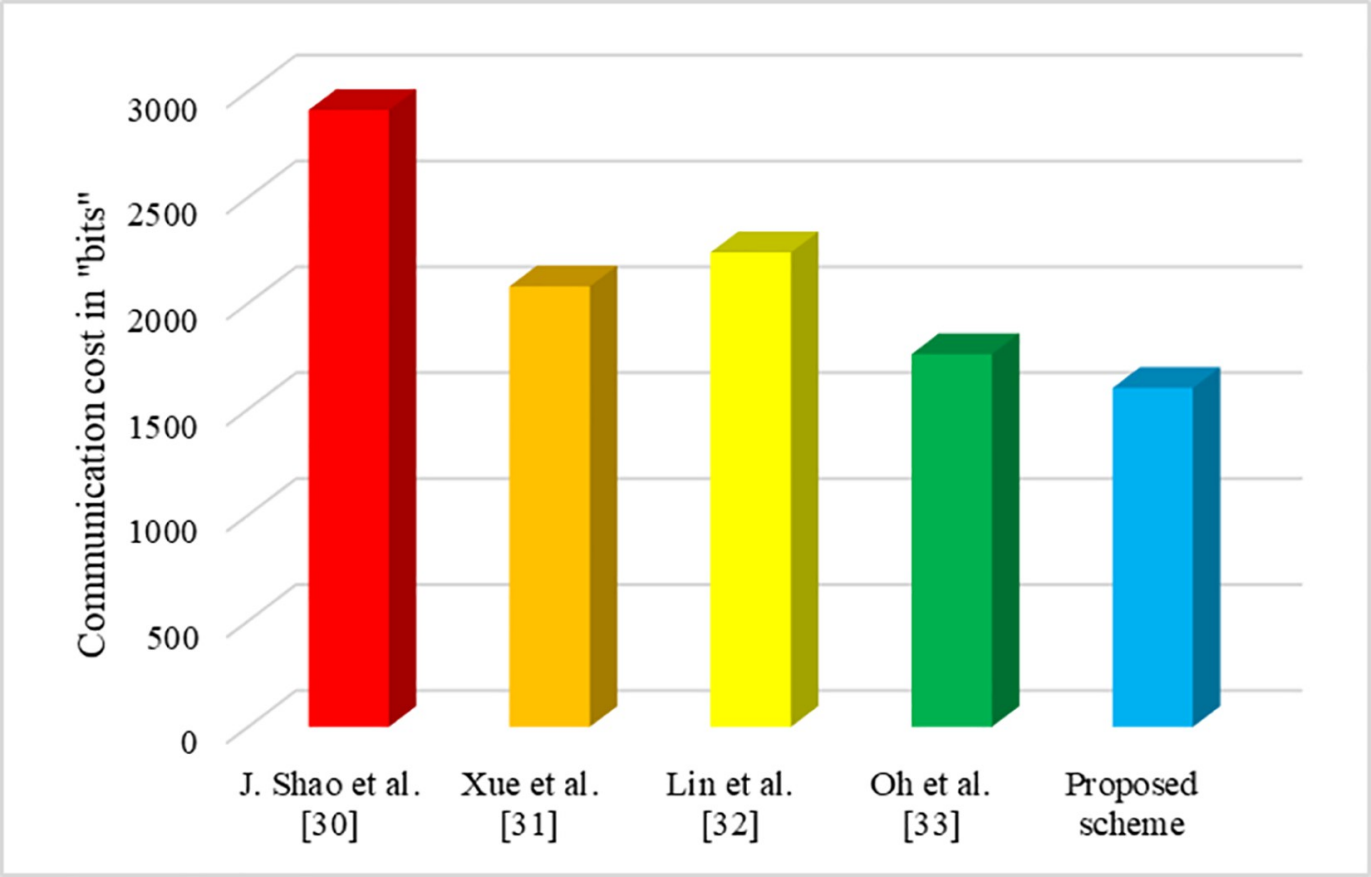
Communication cost of various schemes for single user.

Fig 7 shows the communication cost of various schemes for single user. J. Shao *et al*. [30] requires three messages for the communication and it consumes 2912 bits. Similarly, Xue *et al*. [31] requires four messages for the communication and it consumes 2080 bits. Though, Lin *et al*. [32] and Oh *et al*. [33] schemes require only one and two messages for the communication, but the cost incurred are 2240 bits and 1760 bits. But in our proposed scheme, though two messages are involved for the transformation of information, the communication cost required is only 1600 bits.

**Fig 8:**
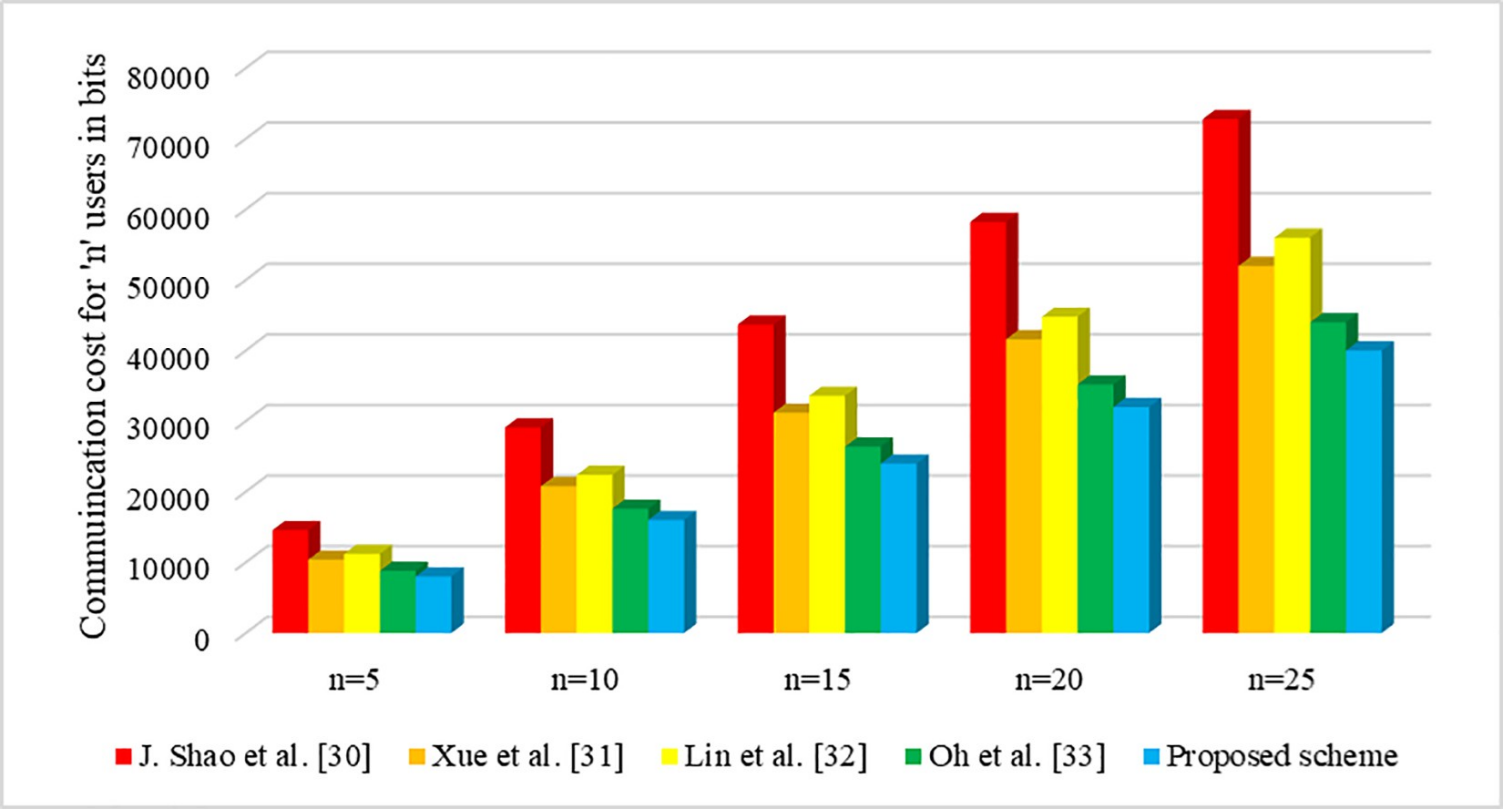
Communication cost of various schemes for ‘*n*’ users.

Similarly, for the *n* number of users, the communication overhead is represented in the Fig 8. Here, the number of user are kept in the range from n=5, 10,15,20 and 25 respectively. Based on the incremental in the number of user, the communication overhead is computed. Moreover, the Fig 8 clearly indicates, as the number of users increases, the communication cost of our suggested work decreases drastically, thus increasing the efficiency of our system. Thus, when compared to the existing schemes, our proposed scheme proved to be noteworthy in terms of communication cost.

### 7.3 Mediator serving capability

Let ℳ be the number of authenticated users who required service from the authenticated mediators. Moreover, ℘ be the probability that the mediator offers service to the authenticated user. ℤ be the total computational time required for verification of signature and certificate. Mediator serving capability is represented by $H=\frac{℘}{M.Z*M}$, where ℤ = 4*T*_*a*_+9*T*_*ex*_+2*T*_*m*_+*T*_*h*_.

**Fig 9:**
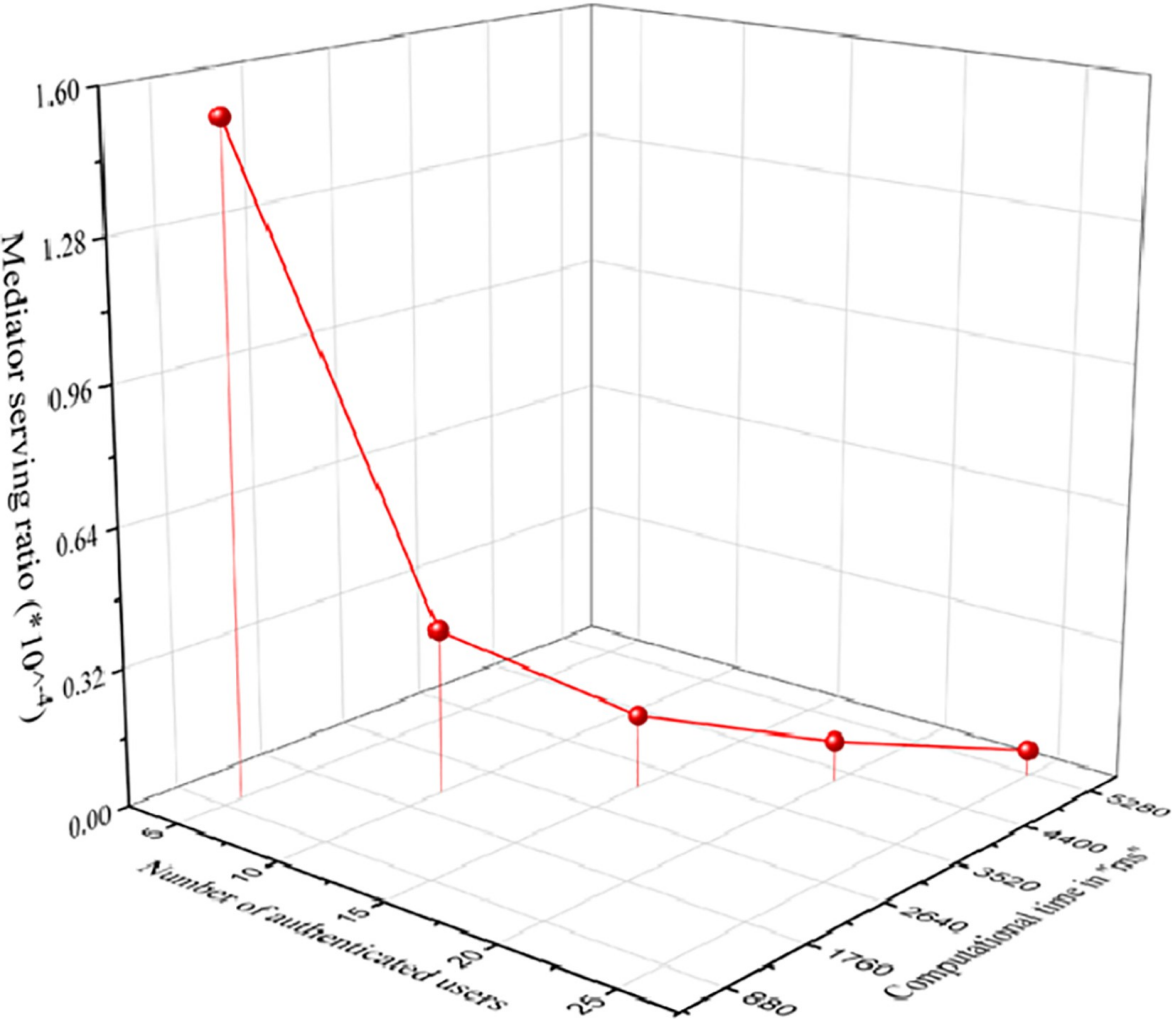
Mediator serving capability.

Fig 9 portrays the graphical representation of mediator serving capability. The graph is plotted between the number of users, total computational cost and the mediator serving capability. From the Fig 9, it is clear as the number of users increases, the computational cost also increases but the service providing capability of the mediator decreases.

## 8. Conclusion

In this work, a trustworthy privacy preserving anonymous authentication scheme is for *OTE* is proposed, allowing both users and mediators to communicate in a secure way. In the proposed TPAAS, user authenticates the mediator in an anonymous way before receiving the message *SM* from the mediator. Similarly, the mediator authenticates the user before receiving the reply from user about the deal. Apart from traditional Online Trading Platforms, our proposed TPAAS provides high level of security, anonymity, privacy to the users and mediators because *OTE* entities use dummy identities, short time keys and signatures to communicate with other entities. Moreover, this scheme allows users to trade with lot of trust by providing conditional privacy and security to them, thereby providing privacy for the genuine users and revoking the privacy of compromised users/mediators. In addition, our proposed scheme is resistant to different kinds of attacks such as Impersonation attack, bogus message attack, message modification attack, Non repudiation attack, Man in the middle attack and Sybil attack. In terms of communication and computation costs, the security and performance analysis revealed that our proposed TPAAS outperforms other traditional schemes. Hence, our scheme satisfies the recent privacy and trust concerns in online trading.

Future work that leads to the incorporation of artificial intelligence that could be used to provide suggestions for the users based on their past shopping experience. Moreover, in the future, auction system for ad exchanges can be included, thereby generating more revenue for the users. In addition, Augmented Reality/ Virtual Reality (AR/VR) technology can be included to allow the users to visualize the products in a comfortable way, allowing them to choose the products that are best suited to them.

The supporting file “S1 Data” refers for the caption as shown in Fig 7: Computational cost for Certificate and signature verification.

## Supporting information

S1 File (DOCX)

S1 Data (XLSX)
