# Supplementary material for: TPAAS: Trustworthy privacy-preserving anonymous authentication scheme for online trading environment
Source: PLoS One. 2024 Nov 18;19(11):e0307738. doi: 10.1371/journal.pone.0307738 (PMC11573216; doi:10.1371/journal.pone.0307738)
Supplement: S1 File — (DOCX) [file pone.0307738.s001.docx]

**Annexure**

***Proof of correctness:***

$H_{i}={UDI}_{i}\times J_{i}=g_{x}^{r+n_{i}+d_{i}}\times g_{x}^{-(n_{i}+d_{i})} =R_{1}$

$I_{i}=D_{i}\times K_{i}=g_{x}^{r+s-d_{i}}\times g_{x}^{d_{i}-r}=S_{1}$

$$\beta_{1}^{'}=\frac{\alpha_{2}^{\beta}.\alpha_{3}^{\beta}.\alpha_{1}^{c_{1}}}{\alpha_{1}^{\beta}{.\alpha}_{2}^{c_{2}}.\alpha_{3}^{c_{3}}}=\frac{\alpha_{2}^{u+k_{j}}.\alpha_{3}^{u+k_{j}}.\alpha_{1}^{k_{j}-x_{1}}}{\alpha_{1}^{u+k_{j}}{.\alpha}_{2}^{k_{j}-x_{2}}.\alpha_{3}^{k_{j}-x_{3}}}=\beta_{1}$$

$$\beta_{2}^{'}=\frac{\alpha_{1}^{\beta}.\alpha_{3}^{\beta}{.\alpha}_{2}^{c_{2}}}{\alpha_{2}^{\beta}{.\alpha}_{1}^{c_{2}}{.\alpha}_{3}^{c_{3}}}=\frac{\alpha_{1}^{u+k_{j}}.\alpha_{3}^{u+k_{j}}.\alpha_{2}^{k_{j}-x_{2}}}{\alpha_{2}^{u+k_{j}}{.\alpha}_{1}^{k_{j}-x_{1}}.\alpha_{3}^{k_{j}-x_{3}}}{=\beta}_{2}$$

$$\beta_{3}^{'}=\frac{\alpha_{2}^{\beta}.\alpha_{1}^{\beta}.\alpha_{3}^{c_{3}}}{\alpha_{3}^{\beta}{.\alpha}_{2}^{c_{2}}{.\alpha}_{1}^{c_{1}}}=\frac{\alpha_{2}^{u+k_{j}}.\alpha_{1}^{u+k_{j}}.\alpha_{3}^{k_{j}-x_{3}}}{\alpha_{3}^{u+k_{j}}{.\alpha}_{2}^{k_{j}-x_{2}}.\alpha_{1}^{k_{j}-x_{1}}}{=\beta}_{3}$$

***Proof of Correctness***

$e\left( sig,N_{j.}g_{y}^{H\left( SM \right)} \right)={e(g}_{x}^{\frac{1}{k_{j}+H\left( SM \right)}},g_{y}^{k_{j}}.g_{y}^{H\left( SM \right)}$) $=e(g_{x}^{\frac{1}{k_{j}+H\left( SM \right)}},g_{y}^{k_{j}+H(SM)}$) $=e(g_{x},g_{y}$) (by using bilinear property)

***Proof of correctness***

$W_{i}={MDI}_{i}\times E_{i}=g_{x}^{r+a_{i}+b_{i}}\times g_{x}^{-(a_{i}+b_{i})}$ $=R_{1}$

$$X_{i}=F_{i}\times B_{i}=g_{x}^{a_{i}+b_{i}}\times g_{x}^{s-a_{i}-b_{i}} =S_{1}$$

$$Q_{i}=P_{i}\times E_{i}=g_{x}^{t+a_{i}+b_{i}}\times g_{x}^{-(a_{i}+b_{i})} =T_{1}$$

$\delta_{1}^{'}=\frac{l_{2}.l_{3}}{l_{1}.l_{5}}$=$\frac{\gamma_{2}^{f_{3}}}{\gamma_{3}^{f_{2}}}.\gamma_{1}^{f_{3}}.\gamma_{2}^{f_{1}}.\frac{\gamma_{3}^{f_{1}}}{\gamma_{1}^{f_{2}}}.\frac{{\gamma_{3}}^{{2f}_{2}}}{{\gamma_{1}}^{{2f}_{3}}}{=\delta}_{1}$

$$\delta_{2}^{'}=\frac{l_{1}.l_{3}}{l_{2}.l_{4}}=\frac{\gamma_{1}^{f_{2}}}{\gamma_{3}^{f_{1}}}.\gamma_{1}^{f_{3}}.\gamma_{2}^{f_{1}}.\frac{\gamma_{3}^{f_{2}}}{\gamma_{2}^{f_{3}}}.\frac{\gamma_{3}^{2f_{1}}}{\gamma_{2}^{2f_{1}}}{=\delta}_{2}$$

$\delta_{3}^{'}={l_{1}.l}_{2}.l_{3}=\frac{\gamma_{1}^{f_{2}}}{\gamma_{3}^{f_{1}}}$.$\frac{\gamma_{2}^{f_{3}}}{\gamma_{3}^{f_{2}}}.\gamma_{1}^{f_{3}}.\gamma_{2}^{f_{1}}{=\delta}_{3}$

***Proof of correctness:***

$e\left( {sig}_{m_{i}},\mu.g_{y}^{H\left( NDI \right)} \right)={e(g}_{x}^{\frac{1}{H\left( NDI \right)+w_{j}}},g_{y}^{w_{j}}.g_{y}^{H\left( NDI \right)}$) $={e(g}_{x}^{\frac{1}{H\left( NDI \right)+w_{j}}},g_{y}^{w_{j}+H(NDI)}$) $=e(g_{x},g_{y}$) (By using bilinear property)

$$\frac{\alpha_{2}^{s}}{\alpha_{1}^{r}}=\frac{{(C}_{i}.R_{1}^{u})^{s}}{(s_{1}^{u})^{r}}=\frac{C_{i}^{s}.g_{x}^{rus}}{g_{x}^{sur}}{=C}_{i}^{s}$$

$\frac{\alpha_{3}^{s}}{\alpha^{s}}$ =$\frac{{(D}_{i}.T_{1}^{u})^{s}}{(T_{1}^{u})^{s}}=\frac{D_{i}^{s}.T_{1}^{us}}{T_{1}^{us}}{=D}_{i}^{s}$

$\frac{\gamma_{2}^{s}}{\gamma_{1}^{t}}=\frac{A_{i}^{s}. T_{1}^{(\frac{f_{1}}{f_{2}}+f_{3})s}}{S_{1}^{(\frac{f_{1}}{f_{2}}+f_{3})t}}= \frac{A_{i}^{s}. T_{1}^{(\frac{f_{1}}{f_{2}}+f_{3})s}}{T_{1}^{(\frac{f_{1}}{f_{2}}+f_{3})s}}$ $= A_{i}^{S}$

$\frac{\gamma_{3}^{t}}{\gamma^{r}}$= $\frac{B_{i}^{t}R_{1}^{(\frac{f_{3}}{f_{1}}+f_{2})t}}{T_{1}^{(\frac{f_{3}}{f_{1}}+f_{2})r}}= \frac{B_{i}^{t}R_{1}^{(\frac{f_{3}}{f_{1}}+f_{2})t}}{R_{1}^{(\frac{f_{3}}{f_{1}}+f_{2})t}}$ $= B_{i}^{t}$
